# Advanced hydrogel therapeutics for intervertebral disc degeneration: Engineering structural–functional properties in natural and synthetic biomaterials

**DOI:** 10.1002/btm2.70059

**Published:** 2025-08-11

**Authors:** Tao Chen, Dading Lu, Siqiao Wang, Huiyi Yang, Wenyong Fan, Zhihui Xiao, Zhaojie Wang, Pooyan Makvandi, Rongrong Zhu, Liming Cheng

**Affiliations:** ^1^ Division of Spine, Department of Orthopedics Tongji Hospital Affiliated to Tongji University School of Medicine Shanghai China; ^2^ Key Laboratory of Spine and Spinal Cord Injury Repair and Regeneration (Tongji University), Ministry of Education School of Medicine, School of Life Science and Technology, Tongji University Shanghai China; ^3^ Department of Neurosurgery Tongji Hospital Affiliated to Tongji University School of Medicine Shanghai China; ^4^ The Quzhou Affiliated Hospital of Wenzhou Medical University, Quzhou People’s Hospital Quzhou Zhejiang China; ^5^ Research and Innovation Cell, Rayat Bahra University Punjab India

**Keywords:** biomaterials, delivery, hydrogel, intervertebral disc degeneration, tissue regeneration

## Abstract

Low back pain is a global health challenge, imposing substantial socioeconomic burdens. Intervertebral disc degeneration (IVDD) is a leading cause of low back pain and can lead to further spinal complications. Current treatments for IVDD are limited to conservative measures and surgery, lacking curative options. Advances in biomaterials science and regenerative medicine have introduced bioactive hydrogels as promising treatments for IVDD. This review summarizes the physiology of the intervertebral disc, the pathophysiology of IVDD, existing diagnostic methods, and current treatment strategies. Importantly, this review examines recent advances in hydrogel treatments for IVDD, emphasizing the biological and material properties of various hydrogels. It compares the advantages and limitations of natural and synthetic hydrogels, outlining the classification of hydrogel delivery substances. Lastly, it identifies current challenges and suggests future research directions to optimize the application of bioactive materials in IVDD treatment.

**Translational Impact Statements**

This review highlights bioactive hydrogels as transformative therapies for IVDD, a key driver of low back pain. Combining biomaterials and regenerative medicine, hydrogels aim to repair discs and prevent degeneration, offering minimally invasive alternatives to current inadequate treatments. By analyzing hydrogel properties and delivery systems, this work advances personalized, clinically viable solutions that could reduce surgical reliance, healthcare costs, and improve global patient outcomes, bridging laboratory innovation to practical care for enhanced quality of life.

**Abbreviations**
AF: annulus fibrosus
ADSC: adipose‐derived stem cell
BMSC: bone marrow mesenchymal stem cell
CEP: cartilage endplate
DTM: decellularized tissue matrix
ECM: extracellular matrix
GelMA: gelatin methacrylamide
GDF5: growth differentiation factor 5
GelMA: gelatin methacrylamide
HA: hyaluronic acid
IVD: intervertebral disc
IVDD: intervertebral disc degeneration
MSC: mesenchymal stem cell
NP: nucleus pulposus
NPC: nucleus pulposus cell
PEG: polyethylene glycol
PRP: platelet‐rich plasma
PBA: polybutyl acrylate
PLGA: poly(lactic‐co‐glycolic acid)
PLLA: polylactic acid
PVA: polyvinyl alcohol
PBA: polybutyl acrylate
PAM: polyacrylamide
ROS: reactive oxygen species
TGF‐β: transforming growth factor‐β

## 1 INTRODUCTION

Low back pain (LBP) is one of the major global public health issues, imposing a significant economic burden on healthcare and social systems.^1^, ^2^ Approximately 70%–85% of individuals experience LBP at some point in their lives. With the increasing aging population, LBP has become one of the primary conditions impacting public health and quality of life, having a profound effect on the socioeconomic landscape.^3^, ^4^ While various factors may contribute to LBP, intervertebral disc degeneration (IVDD) is the most common cause.^5^ Characterized by structural and metabolic changes, IVDD is a chronic, complex, and multifactorial musculoskeletal disease that gradually results in the loss of mechanical stability and shock‐absorbing function of the intervertebral disc (IVD).^6^ However, the pathogenesis and progression of IVDD are intricate processes, and its specific mechanisms remain incompletely understood. Currently, there are only conservative treatments and surgical interventions available for patients with IVDD, with no drugs or methods capable of curing the condition.^7^ In this context, bioactive materials that facilitate the regeneration and repair of the musculoskeletal system are emerging as promising new options for treating spinal degenerative diseases.

This review offers a concise overview of the physiology of IVD and the pathophysiology of IVDD, summarizing its diagnostic methods and current treatment options. Importantly, it examines the recent advancements in hydrogels for treating IVDD and highlights the biological and material properties of various types of hydrogels. Additionally, it outlines the advantages and disadvantages of both natural and synthetic hydrogels and summarizes the classification of hydrogel delivery substances. Finally, the review addresses the challenges facing this field and outlines future directions aimed at enhancing the application of bioactive materials in IVDD treatment.

## 2 THE PHYSIOLOGY OF IVD

Composed of the peripheral annulus fibrosus (AF), the central nucleus pulposus (NP), and the cartilage endplate (CEP), the IVD is a cartilaginous structure located between two adjacent vertebrae. It functions as a shock absorber by distributing mechanical stress along the spine and facilitating trunk movement.^8^

The NP serves as the primary functional element of the IVD, consisting of water, nucleus pulposus cells (NPC), and extracellular matrix (ECM). Under normal physiological conditions, NPCs generate a well‐hydrated ECM abundant in type II collagen and proteoglycan.^9^ Their role is to preserve moisture and enable the IVD to withstand compressive forces, achieved through the hydraulic distribution within the IVD.^10^

The AF can be divided into two regions: the outer AF, primarily composed of organized type I collagen fibers and characterized by high tensile strength, and the inner AF, which serves as a transitional zone between the outer AF and NP, with lower density and weaker organization.^11^ The primary function of the AF is to counteract and maintain the osmotic pressure exerted by the NP through its tensile strength, providing natural resistance to bending, torsion, and shear, particularly during flexion, extension, and rotational movements of the IVD.^12^

The CEP interfaces with the adjacent vertebrae and consists of hyaline cartilage cells and chondrocytes. It can endure significant loads during spinal movements and helps distribute the internal pressure of the IVD to the neighboring vertebrae.^13^ As the largest avascular tissue in the human body, the IVD relies on the CEP as the primary conduit for glucose and oxygen to reach the IVD cells through the vertebral capillaries. Additionally, the CEP plays a crucial role in the removal of waste products from the IVD.^14^

## 3 THE PATHOPHYSIOLOGY OF IVDD

IVDD is a multifactorial degenerative disease influenced by various factors, including aging, genetics, mechanical load, biological rhythms, nutrition, and environmental elements.^15^ This condition can result in symptoms such as neck pain, back pain, and limited spinal mobility. In severe cases, it may compress nerve roots or the spinal cord, leading to radicular pain or neurological dysfunction.^16^ Commonly encountered spinal disorders in clinical practice, such as cervical spondylosis, lumbar disc herniation, and lumbar spinal stenosis, are often closely linked to IVDD.^17^ Notably, IVDD detected by imaging (e.g., MRI findings like disc height loss, signal changes, and Pfirrmann grading) is common with aging but does not always cause symptomatic discogenic pain. Diagnosing discogenic pain (pain originating from degenerated discs) remains challenging and requires both: (1) correlation between IVDD imaging features and clinical symptoms, and (2) exclusion of other pain sources. These symptoms may arise from IVD degeneration at molecular, cellular, and structural levels.

The imbalance between the anabolism and catabolism of NP plays a crucial role in IVDD. During this process, NPCs experience aging, resulting in reduced synthesis of proteoglycans and a shift from type II to type I collagen.^18^ This transition leads to decreased ECM synthesis. Moreover, as degeneration advances, NPCs abnormally secrete matrix metalloproteinases (MMP)‐1, MMP‐3, and other matrix‐degrading enzymes, which accelerate the breakdown of proteoglycans and type II collagen, thereby increasing ECM degradation.^19^, ^20^ Consequently, the decrease in water content of the NP and the reduction in disc height ultimately result in a diminished ability of the IVD to buffer mechanical loads.

The AF cells undergo apoptosis, leading to a reduction in cell number. These cells also abnormally secrete MMPs, which accelerate the degradation of collagen and other matrix components. The layered structure of the AF gradually deteriorates, with collagen fibers becoming irregular, loosely arranged, and even fractured. The collagen in the inner layers of the AF transitions from fibrous to cartilaginous, compromising the integrity and tensile strength of the AF and facilitating the herniation of the NP through the AF.^12^, ^21^ Additionally, neovascularization occurs, with microvessels growing into the inner layers of the AF and even into the NP, affecting the nutritional supply to the IVD.^22^

**FIGURE 1:**
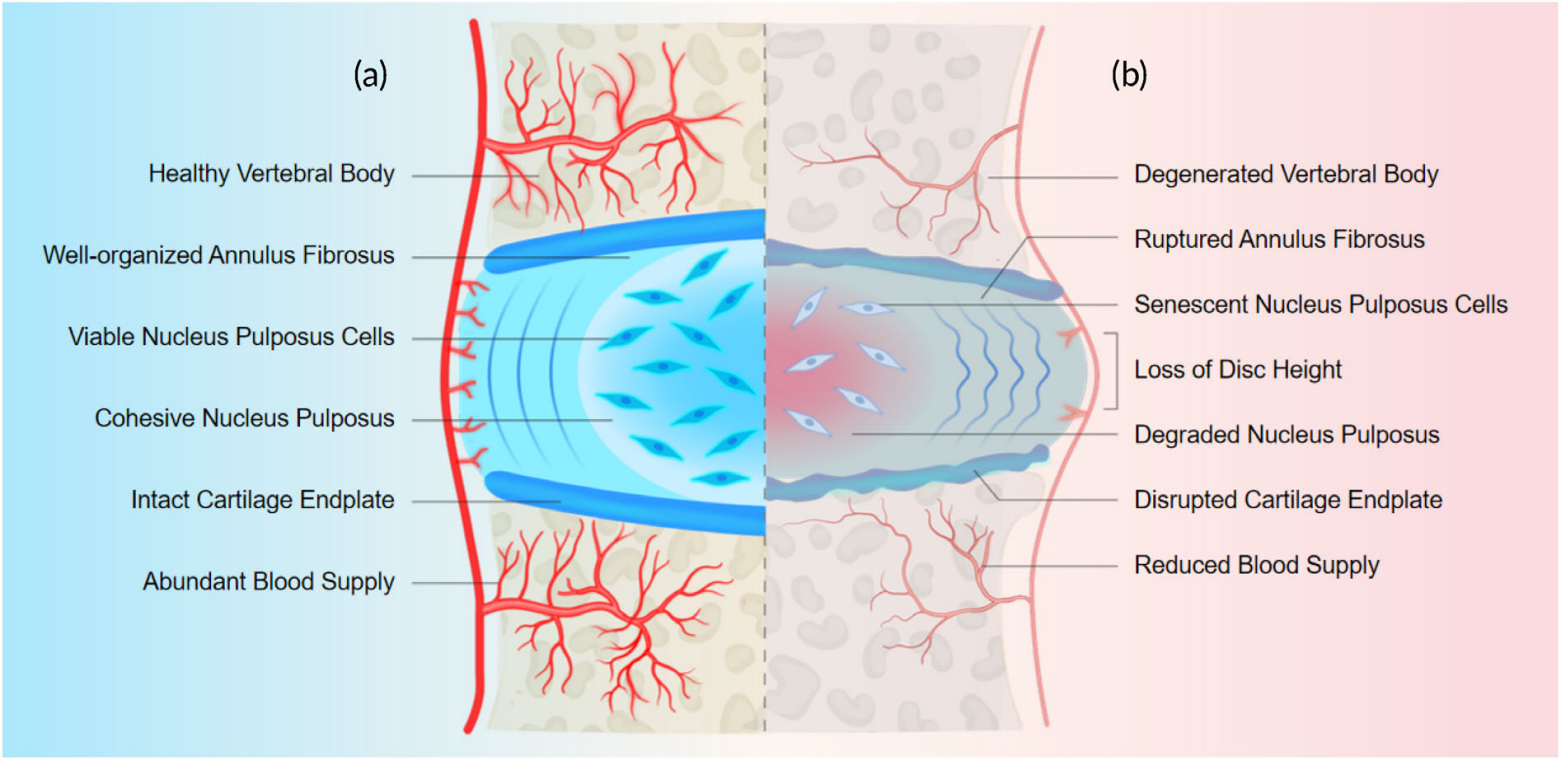
Schematic illustration contrasting the healthy and degenerated intervertebral disc. (a) Healthy vertebral body, well‐organized annulus fibrosus, viable nucleus pulposus cells, cohesive nucleus pulposus, intact cartilage endplate, and abundant blood supply. (b) Degenerated vertebral body, ruptured annulus fibrosus, senescent nucleus pulposus cells, loss of disc height, degraded nucleus pulposus, disrupted cartilage endplate, and reduced blood supply. Created with www.BioRender.com.

Chondrocytes in the CEP undergo apoptosis, leading to a reduction in cell number. The remaining chondrocytes exhibit diminished functionality, impairing their ability to effectively maintain and repair the cartilage matrix.^23^ Moreover, changes in the proportions and distribution of matrix components affect the mechanical properties and elasticity of the CEP. Collagen fibers become loosely arranged and irregular. The CEP may also thin, reducing its load‐bearing capacity. Furthermore, calcification or ossification of the CEP can occur, resulting in a stiffened connection between the CEP and the IVD^24^, ^25^ (Figure 1).

## 4 DIAGNOSIS AND CURRENT TREATMENTS OF IVDD

The diagnosis of IVDD is primarily based on the clinical symptoms and signs. It is then confirmed through supplementary imaging analysis (such as X‐ray, CT scan, or magnetic resonance imaging (MRI)) and the severity of degeneration is assessed using specific scoring systems.^26^, ^27^, ^28^ Currently, there are two common methods for treating IVDD: conservative treatment (including non‐drug treatments and drug treatments) and surgical treatment.^29^

For patients with mild IVDD, maintaining a healthy lifestyle, engaging in regular exercise, and undergoing appropriate physical therapy are effective and straightforward non‐pharmacological treatment options.^30^ In terms of pharmacological treatment, commonly used medications include analgesics, opioid pain relievers, nonsteroidal anti‐inflammatory drugs (NSAIDs), muscle relaxants, and steroid drugs. These medications are intended to manage pain, improve physical function, and enhance quality of life. However, they can have side effects, and there is a risk of some patients developing dependency on them.^31^, ^32^ In the early stages of the condition, physical therapy, analgesics, and anti‐inflammatory drugs can provide temporary relief from mild symptoms. In more advanced stages, localized steroid injections may be used to address radicular symptoms.^17^, ^33^

**FIGURE 2:**
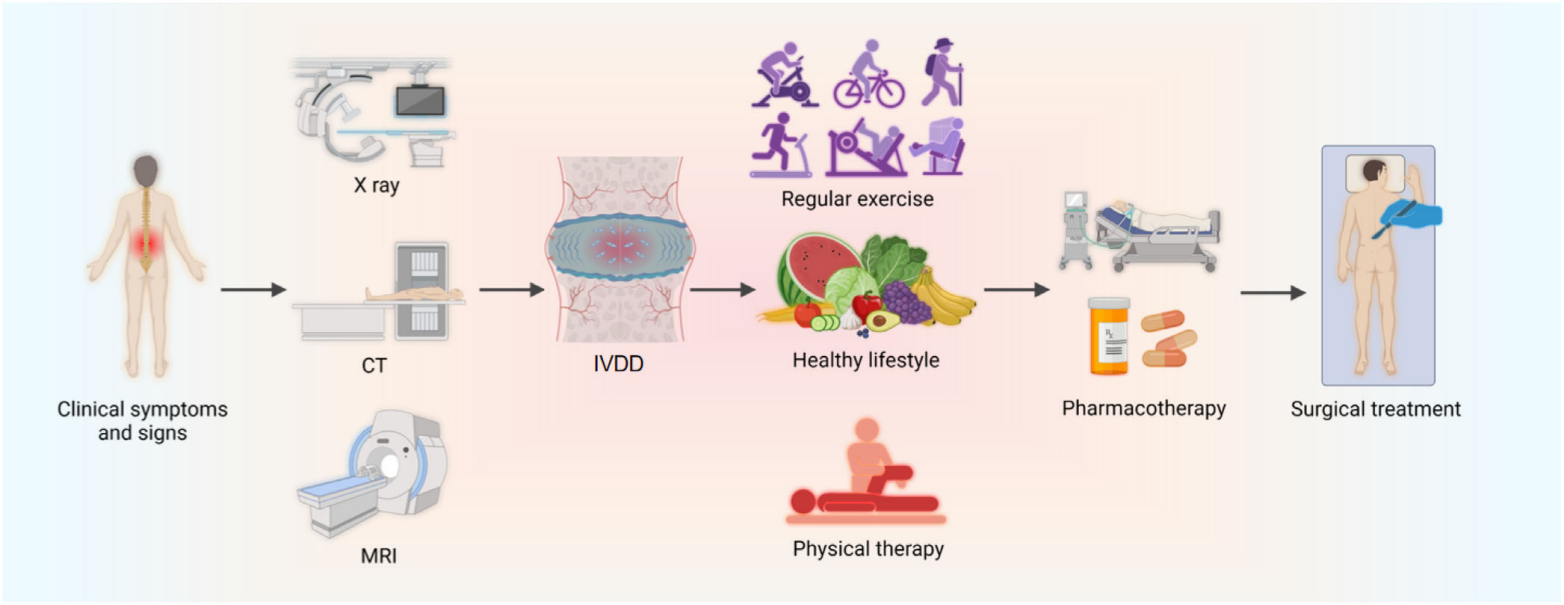
Intervertebral disc degeneration diagnosis and treatment illustration. Diagnosis: IVDD is diagnosed through the comprehensive assessment of clinical symptoms, physical signs, and imaging studies. Treatment: (i) regular exercise, healthy lifestyle, physical therapy; (ii) pharmacotherapy; and (iii) surgical treatment (CT, computed tomography; IVDD, intervertebral disc degeneration; MRI, magnetic resonance imaging). Created with www.BioRender.com.

If conservative treatments fail or any of the following conditions occur, surgical intervention may be required: severe radicular symptoms, acute nerve root compromise, cauda equina syndrome, or spinal instability. Surgical options include discectomy, spinal fusion, and total disc replacement. Discectomy may be performed as a partial or total procedure.^17^, ^34^ Partial discectomy is a minimally invasive technique that removes protruding IVD tissue under microscopic guidance, while total discectomy involves the complete removal of the damaged IVD, often combined with spinal fusion.^35^ Spinal fusion entails fusing or connecting two adjacent vertebrae and is considered the gold standard for treating IVDD.^36^ Total disc replacement involves replacing the degenerated IVD with an artificial one, typically used for single‐level disc degeneration.^37^ While surgery can relieve clinical symptoms, it may increase the risk of degeneration at the surgical site or adjacent segments, and there is a possibility of pain recurrence^38^, ^39^, ^40^ (Figure 2).

In summary, while pharmacological and surgical treatments can improve clinical symptoms in patients, their long‐term effectiveness remains only moderate and somewhat uncertain. Medications provide symptom relief but do not offer a complete cure for IVDD. Surgery can fully remove the degenerated IVD segment but may lead to worsening degeneration in adjacent segments.^41^, ^42^ Consequently, considering the physiology of IVD and the pathophysiology of IVDD, there is a need for more optimal and valuable treatment strategies to prevent, delay, or even reverse the progression of IVDD.

## 5 BENEFITS OF HYDROGELS FOR THE TREATMENT OF IVDD

Hydrogel is a material known for its high water content and three‐dimensional network structure, which enables it to absorb substantial amounts of water and form a gel‐like substance.^43^, ^44^ Hydrogels are extensively used in medicine, drug delivery, and tissue engineering, offering several notable advantages, especially in treating IVDD: (1) mechanical properties: in situ cross‐linked hydrogels, when injected locally, exhibit great mechanical properties and plasticity. They can effectively restore IVD height, bear mechanical loads, and support spinal movement; (2) biocompatibility: most hydrogels are highly biocompatible, showing minimal toxicity to tissues or foreign cells and safeguarding bioactive molecules from local degradation; (3) biodegradability: most hydrogels possess favorable biodegradability, with their degradation products being non‐toxic and safely metabolizable by the body; (4) mimicking the natural NP: since the NP is a tissue with high water content, hydrogels, which also have high water content, are especially well‐suited for strategies aimed at IVD regeneration.^45^, ^46^, ^47^ This review highlights the potential of hydrogels to address IVDD's structural and biochemical consequences. Their primary objective is to restore disc structure/function, potentially slowing degeneration and creating a less pain‐conducive environment. While pain relief remains the ultimate clinical goal, these biomaterials directly target radiologically confirmed degenerative pathology rather than the complex neurophysiology of pain perception.

## 6 MAJOR TYPES OF HYDROGELS FOR THE TREATMENT OF IVDD

In recent years, biopolymer hydrogels have garnered significant attention for the treatment of IVDD, primarily due to their diversity and unique characteristics. Natural hydrogels, such as gelatin, alginate, and collagen, are known for their great biocompatibility and biodegradability, providing high‐quality support and nourishment to IVD. Similarly, synthetic hydrogels like polyethylene glycol and polylactic acid have emerged as popular research topics because of their tunable physicochemical properties and controllable degradation rates. These diverse hydrogels optimize mechanical properties and release characteristics through various crosslinking methods and modification techniques.^48^, ^49^ Furthermore, the development of composite hydrogels that combine natural and synthetic materials not only enhances mechanical strength but also improves biological functionality. As a result, these various biopolymer hydrogels present new ideas and possibilities for treating IVDD, significantly expanding their clinical application prospects.^50^, ^51^

**FIGURE 3:**
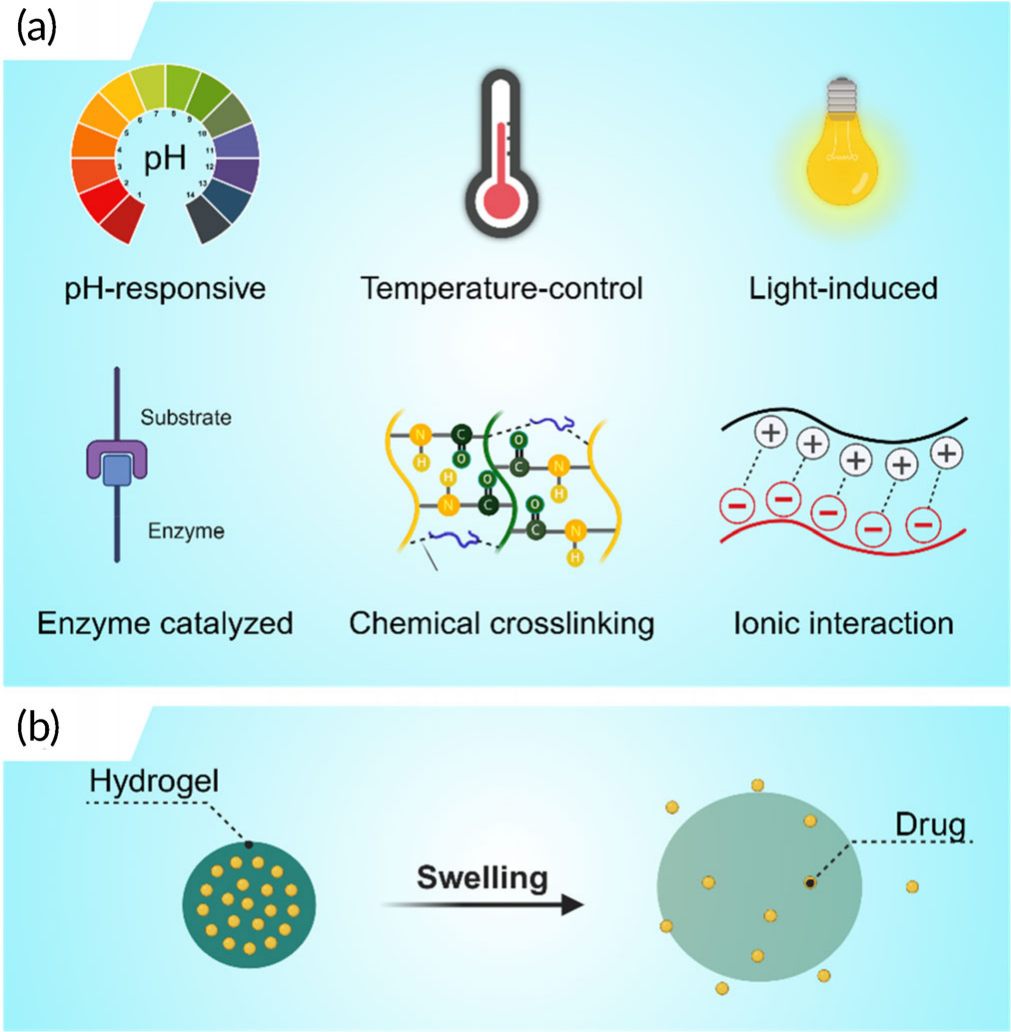
Schematic illustration of hydrogels cross‐linking methods and drug release. (a) The methods of hydrogel crosslinking: pH‐responsive, temperature‐controlled, light‐induced, enzyme‐catalyzed, chemical crosslinking, and ionic interaction. (b) Drug release mediated by swelling‐triggered network expansion and enhanced diffusion. Created with www.BioRender.com.

Building upon the diverse landscape of biopolymer hydrogels for IVDD treatment, recent research has extensively explored a wide array of injectable formulations. These include natural hydrogels (e.g., hyaluronic acid, collagen/gelatin, alginate, chitosan, polypeptides, fibrin, and chondroitin sulfate; see Table 1), synthetic hydrogels (e.g., gelatin methacrylate, polyethylene glycol, polycaprolactone, polylactic acid/polyglycolic acid, polyvinyl alcohol, and polyacrylamide; see Table 2), and ECM hydrogels. Gelation of these hydrogels is achieved through various mechanisms, broadly categorized as pH‐responsive, temperature‐controlled, photo‐induced, enzyme‐catalyzed, chemical crosslinking, and ionic interactions (Figure 3). In the following sections, we will systematically evaluate the composition, biological properties, and material characteristics of these representative hydrogel types.

**TABLE 1:** Recent advances in natural hydrogels for IVDD treatment.

Category	Components	Biological properties	Material properties	Ref
Hyaluronic acid	HA/PEG	Reduce nociceptive behavior, anti‐inflammatory	Hydrophilic, biocompatibility	^55^
	HA/M2‐exosome	Promote ECM generation	Good encapsulation, biocompatibility	^47^
	HA/chitosan/dopamine	Anti‐inflammatory, antioxidant, promote ECM generation	pH‐responsive, good mechanical properties	^56^
	HA/gefitinib	Activate autophagy, promote ECM generation	Temperature sensitive, controlled release	^57^
	HA/polyamide amine/siRNA	Anti‐inflammatory, promote tissue repair	Self‐healing, controlled release, biocompatibility	^58^
	HA/ZrO_2_	Promote ECM generation	Visualization, slow degradation, matching mechanical properties	^59^
	HA/BMSC/salvianolic acid B	Inhibit BMSC apoptosis	Biocompatibility, hygroscopicity	^60^
	HA/methylcellulose/MSC	Maintain cell viability, promote cell phenotypic maintenance	Controlled release, biocompatibility	^61^
	HA/collagen/riboflavin/ BMSC/FG4592	Promote BMSC proliferation and differentiation, promote ECM generation	Rapid gelation, biodegradability, good mechanical properties	^62^
	HA/silk fibroin	Promote stem cell differentiation	Sustained release	^63^
	HA/liposome	Promote ECM generation	Biodegradability, sustained release, hygroscopicity	^64^
	HA/MnO_2_/lactate oxidase	Anti‐inflammatory, promote ECM generation	Controlled release, biocompatibility	^65^
Collagen/gelatin	Collagen	Promote ECM generation, promote cell migration	3D spheroid‐forming capability, water‐solubility, biodegradability, low antigenicity	^68^
	Collagen/HA	Maintain moisture	Biocompatibility, great mechanical properties	^69^
	Collagen/HA	Relieve pain, maintain moisture	Shape memory, hygroscopicity	^70^
	Collagen	Promote ECM generation	Maintains disc height	^71^
	Gelatin/kartogenin	Promote ECM generation, antioxidant	Self‐healing	^74^
	Gelatin/β‐cyclodextrin/melatonin/Au	Maintain moisture, promote ECM generation	Biocompatibility, self‐healing	^75^
	Gelatin/borax/oxidized HA/prussian blue nanoparticle	Antibacterial, antioxidant	Self‐healing, good mechanical properties	^76^
	Gelatin/MSC	Promote MSC differentiation, promote ECM generation	Shear‐thinning, self‐healing, moldability, biocompatibility, biodegradability	^77^
	Gelatin/PRP/simvastatin	Anti‐inflammatory, promote tissue repair	Biocompatibility, hygroscopicity, sustained release	^78^
	Gelatin/tetrazine/norbornene/TGF‐β	Promote cell adhesion, promote ECM generation, relieve pain, maintain moisture	Rapid gelation, biodegradability, sustained release	^79^
	Gelatin/PBA/polyamide amine/oxidized dextran/siRNA/	Anti‐inflammatory, antibacterial, gene therapy	Sustained release, viscoelasticity, self‐healing	^80^
Alginate	Sodium alginate/gelatin/thermoplastic urethane	Scavenge ROS, promote ECM generation, promote cell proliferation	ROS‐responsive, good resilience, biocompatibility	^82^
	Alginate/gelatin/curcumin/ polylactic acid nanoparticle	Anti‐inflammatory, promote cell proliferation, immune regulation	Good encapsulation, biocompatibility, hygroscopicity, good mechanical properties	^83^
	Sodium alginate/gelatin/Zn^2+^/antagomir	Antibacterial, inhibiting cell apoptosis, promote ECM generation, maintain moisture	Great mechanical properties, biocompatibility, biodegradability, hygroscopicity, sustained release	^84^
	Sodium alginate/mesoporous bioactive glasses/melatonin	Anti‐inflammatory, antioxidant	Rapid gelation, sustained release, good mechanical properties	^85^
	Alginate/BMSC/NPC	Promote BMSC differentiation, promote ECM generation	High water retention, biocompatibility	^86^
	Alginate/gelatin/MSC	Inhibit cell pyroptosis, promote MSC migration, proliferation, and differentiation	Good encapsulation, biocompatibility	^87^
	Alginate	Promote cell proliferation	Biocompatibility	^88^
	Alginate/integrin/syndecan	Promote cell adhesion, proliferation	Interpenetrating network, biocompatibility	^89^
	Sodium alginate/poly(N‐isopropyl acrylamide)/ silicate ceramics/Mg^2+^/Ca^2+^/NPC	Induction of macrophage M2 polarization, promote ECM generation	Interpenetrating network, rapid gelation, good encapsulation	^90^
Chitosan	Chitosan/glycerophosphate/ MnOx	Scavenge ROS, maintain cell viability, promote BMSC differentiation	Temperature sensitive, biocompatibility	^93^
	Chitosan/celecoxib	Anti‐inflammatory	Biocompatibility, biodegradability	^94^
	Chitosan/gelatin/laponite/celecoxib/layered double hydroxide	Promote cell proliferation and ECM generation, anti‐inflammatory	Self‐healing, rapid gelation, biocompatibility	^95^
	Chitosan/HA/hydroxyapatite nanorod/epigallocatechin‐3‐gallate	Induction of macrophage M2 polarization, regulate ECM metabolism	Sustained release, self‐healing	^98^
	Chitosan/antagomir‐21/tannic acid nanoparticle	Anti‐inflammatory, antioxidant, promote ECM generation	pH‐responsive, sustained release, biocompatibility, biodegradability	^99^
	Chitosan/decellularized matrix/GDF5/PLGA	Promote stem cell differentiation, promote ECM generation	Sustained release, biomimetic mechanics	^100^
Polypeptide	Active peptide APETx2/GelMA/NPC	Anti‐inflammatory, regulate the balance of ECM metabolism	Promote ECM generation, biocompatibility	^104^
	Laminin‐mimetic peptides/PEG/NPC	Enhance cell viability, promote cell phenotypic maintenance	In vivo stability, biocompatibility	^105^
	Octapeptide FEFKFEFK/graphene oxide/NPC	Anti‐inflammatory, promote ECM generation	Biocompatibility	^106^
	Polypeptide RAD/SA1	Promote ECM generation	Biocompatibility	^107^
	Arginine–glycine–aspartic acid tripeptide/DTM/extracellular vesicles	Anti‐senescence, promote cell migration, alleviate cell cycle arrest	Prolonged extracellular vesicles retention, biocompatibility	^108^
	Polypeptide/MnO_2_ nanosheet/ GDF‐5	Relieve pain, inhibit immune response, promote ECM remodeling, antioxidant	Biodegradability, biocompatibility	^109^
Fibrin	Fibrin–Genipin/poly(trimethylene carbonate)/polyurethane	Promote cell adhesion and proliferation, promote tissue generation	Biomimetic mechanics, biocompatibility, tissue adhesion	^112^
	Fibrin/alginate/annulus fibrosus cell	Inhibit cell apoptosis, promote ECM generation	Good mechanical properties, reduce herniation risk	^113^
	Fibrin/fibronectin/PEG/glycosaminoglycan	NA	Interpenetrating network, great adhesion, good sealing	^114^
	Fibrin–Genipin/collagen	Anti‐inflammatory, promote cell adhesion and proliferation	Biocompatibility, sustained release	^115^
	Silk fibrin/sodium alginate/PRP	Promote cell proliferation	Good compressive strength, sustained release	^116^
	Fibrin/kaempferol	Regulate the balance of ECM metabolism, anti‐inflammatory	Sustained release, biocompatibility	^117^
	Fibrin/extracellular vesicles	Rectify aberrant fatty acid metabolism, inhibit cell pyroptosis, anti‐inflammatory	Stability, sustained release, biocompatibility, biodegradability	^118^
Chondroitin sulfate	Chondroitin sulfate/collagen/ADSC	Promote ADSC differentiation	Biocompatibility	^121^
	Chondroitin sulfate/gelatin/dopamine/extracellular vesicles/ Glutaredoxin3	Reduce mitochondrial damage, scavenge ROS	Sustained release, biodegradability	^122^
	Chondroitin sulfate/ECM	Promote ECM generation	Tissue adhesion, biocompatibility	^123^

Abbreviations: ADSC, adipose‐derived stem cell; BMSC, bone marrow mesenchymal stem cell; DTM, decellularized tissue matrix; ECM, extracellular matrix; GDF5, growth differentiation factor 5; GelMA, gelatin methacrylamide; HA, hyaluronic acid; MSC, mesenchymal stem cell; NPC, nucleus pulposus cell; PEG, polyethylene glycol; PRP, platelet‐rich plasma; PBA, polybutyl acrylate; PLGA, poly(lactic‐co‐glycolic acid); ROS, reactive oxygen species; TGF‐β, transforming growth factor‐β.

**TABLE 2:** Recent advances in synthetic hydrogels for IVDD treatment.

Category	Components	Biological properties	Material properties	Ref.
GelMA	GelMA/vanillin/TGFβ3	Anti‐inflammatory, antioxidant, promote ECM generation	Biocompatibility, sustained release, high water retention	^126^
	GelMA/TGF‐β/catalase	Neutralize the acidic microenvironment, anti‐inflammatory, promote ECM generation	Good encapsulation, biocompatibility	^127^
	GelMA/aspirin/liposome	Anti‐inflammatory, promote cell adhesion and proliferation	Photo‐crosslinking, sustained release, biocompatibility	^128^
	GelMA/polydopamine/diclofenac sodium	Anti‐inflammatory, anti‐apoptotic, promote ECM generation	Photothermal effect, controlled‐release	^129^
	GelMA/epigallocatechin‐3‐gallate	Promote cell phenotypic maintenance, anti‐inflammatory, antioxidant	ROS responsive, biocompatibility, biodegradability	^130^
	GelMA/fucoidan	Promote cell phenotypic maintenance, scavenge ROS, promote ECM generation	High water retention, sustained release, biocompatibility	^131^
	GelMA/HA/luteolin	Tissue adhesion, antioxidant, antibacterial	pH‐responsive, ROS responsive, biocompatibility	^132^
	GelMA/MnO_2_	Antioxidant, induction of cellular autophagy	Biocompatibility, porous structure	^133^
	GelMA/chitosan/black phosphorus quantum dot	Antioxidant, anti‐inflammatory, promote ECM generation	Good encapsulation, sustained release, biocompatibility	^134^
	GelMA/HA/histidine/Mg^2+^	Promote cell adhesion and proliferation, scavenge ROS, anti‐inflammatory, anti‐senescence	Sustained release, biocompatibility	^135^
PEG	PEG/chitosan	Promote tissue repair, promote ECM generation	Strong compressive strength, rapid gelation, biocompatibility, good sealing	^138^
	PEG/miR‐29/cationic block copolymer	Suppress fibrosis	Responsive release	^139^
	PEG/Agomir874/Ag^+^	Antibacterial, regulate the balance of ECM metabolism	Self‐healing, biodegradability, hygroscopicity	^140^
	PEG/PLGA/bevacizumab	Promote ECM generation	Biocompatibility, sustained release	^141^
	PEG/PLGA/MR409	Inhibit secretory autophagy, anti‐inflammatory, antioxidant	ROS responsive, temperature sensitive, viscoelasticity, sustained release	^142^
	PEG/PRP	NA	Sustained release	^143^
PCL	PCL/celecoxib	Anti‐inflammatory, promote ECM generation	Sustained release	^146^
	PCL/chitosan/perfluorotributylamine	Promote cell migration and proliferation, promote ECM generation	Release oxygen, interpenetrating network, biocompatibility	^147^
	PCL/HA	Optimize matrix distribution	Biomimetic mechanics, structural mimicry, slow degradation	^148^
	PCL/HA	Maintain ECM components	Biomimetic structure	^149^
	PCL/polyethylene oxide	Promotes cell colonization, optimize matrix distribution	Interpenetrating network, structural mimicry	^150^
	PCL	Promote cell adhesion, promote ECM deposition, repair annulus fibrosus defect	Tissue adhesion, good sealing, uniformly aligned fibers, biomimetic mechanics	^151^
PLLA/PLGA	PLLA/HA/TGF‐β3/ibuprofen	Promote ECM generation, anti‐inflammatory	Sustained release, biomimetic mechanics, good sealing	^153^
	PLLA/PLGA/chitosan/chitosan	Promote cell adhesion	Interpenetrating network, biocompatibility	^154^
	PLGA/GelMA/PRP/kartogenin	Promote ADSC differentiation, antioxidant	Biocompatibility, sustained release	^156^
PVA	PVA/glycerol	Regulate the balance of ECM metabolism, promote cell proliferation	Viscoelasticity, energy‐dissipating capability, biomimetic mechanics	^158^
	PVA/PBA/melatonin/liposome	Activate circadian clock, scavenge ROS, promote ECM generation	Good encapsulation, controlled release	^159^
	PVA/PCL/MOF/histidine/Zn^2+^	Anti‐inflammatory, promotes ECM secretion and cell migration, scavenge ROS	ROS‐responsive, sustained release	^161^
	PVA/tsPBA/SLC7A11	Inhibit ferroptosis, scavenge ROS	Good encapsulation, ROS‐responsive, controlled release, biocompatibility	^162^
	PVA/tsPBA/rapamycin	Scavenge ROS, anti‐inflammatory	Controlled release, biocompatibility	^163^
PAM	PAM/polyacrylic acid/polydopamine	Promote cell adhesion, promote ADSC differentiation	Reactive expansion, static stretch, biocompatibility	^165^
	PAM/dopamine/HA/collagen mimetic peptide/TGF‐β1	Promote cell phenotypic maintenance, anti‐inflammatory, recruit cell, promote ECM generation	High compressive strength, tissue adhesion, self‐healing, good sealing	^166^
	PAM/alginate/chitosan	Promotes cell survival and matrix deposition	Viscoelasticity, good sealing, matching mechanical strength	^167^
ECM	ECM/ADSC	Promote tissue generation	Biomimetic mechanics, slow degradation	^173^
	ECM/stem cell/Lenti‐Sphk2	Anti‐senescence, promote autophagy	Good encapsulation, sustained release	^174^
	ECM/ADSC	Gene induction, promote ADSC differentiation	Matching mechanical strength, biocompatibility	^175^
	ECM	Promote BMSC differentiation, promote tissue regeneration	Biocompatibility	^176^
	ECM/extracellular vesicles	Inhibit cell pyroptosis, promote cell migration	Temperature sensitive, biocompatibility, controlled release, good encapsulation	^177^
	ECM/extracellular vesicles/Pluronic F127	Promote cell proliferation and migration, promote ECM generation	Temperature sensitive, biocompatibility	^178^
	ECM/exosome	Regulate the balance of ECM metabolism, anti‐inflammatory	Temperature sensitive, in situ gelation	^179^
	ECM/lactate oxidase/MnO_2_/nanozyme	Lactic acid consumption, promote autophagy, promote ECM generation	High water retention, sustained release	^180^
	ECM/cationic nanoparticle/TrkA‐IN‐1	Inhibit nerve ingrowth and sensitization, anti‐hyperalgesic, anti‐inflammatory	Sustained release, biocompatibility	^181^

Abbreviations: ADSC, adipose‐derived stem cell; BMSC, bone marrow mesenchymal stem cell; ECM, extracellular matrix; GelMA, gelatin methacrylamide; HA, hyaluronic acid; MR409, growth hormone‐releasing hormone analog; PAM, polyacrylamide; PBA, polybutyl acrylate; PEG, polyethylene glycol; PLGA, poly(lactic‐co‐glycolic acid); PLLA, polylactic acid; PRP, platelet‐rich plasma; PVA, polyvinyl alcohol; ROS, reactive oxygen species; SLC7A11, solute carrier family 7 member 11; TGF‐β, transforming growth factor‐β.

### 6.1 Natural hydrogels

#### 6.1.1 Hyaluronic acid

Hyaluronic acid (HA) is a naturally occurring polysaccharide found in human tissues, renowned for its exceptional moisturizing, viscoelastic, filling, and repairing properties.^52^ Its applications are extensive, and in the context of IVDD, (1) HA absorbs and retains moisture within the IVD; (2) HA enhances tissue deformability and reduces internal friction within IVD components during mechanical loading; (3) HA offers structural support within the IVD, helping to maintain its structure and elasticity; and (4) the HA‐based biological scaffold supports cell growth and tissue regeneration.^53^, ^54^ In addition to the common applications mentioned, HA hydrogels also demonstrate unique potential in pain relief. Isa et al. innovatively designed a novel rat model of pain induced by IVD injury and evaluated the efficacy of HA hydrogels in alleviating pain. The results indicate that HA hydrogels alleviate pain by modulating glycosylation changes, reducing the activity of key inflammatory signaling molecules, and regulating protein pathways, which in turn inhibit the expression of pain markers and effectively reduce pain perception.^55^

To regulate ECM metabolism and preserve IVD structure, Liu et al. developed an HA hydrogel incorporating M2c macrophage exosomes, demonstrating its ability to modulate ECM metabolic balance through the miR‐124/CILP/TGF‐β regulatory axis.^47^ Similarly, Luo et al. designed a multifunctional HA hydrogel with injectability, antioxidant properties, and anti‐inflammatory capabilities to regulate ECM metabolism. This hydrogel, which matches the mechanical properties of the human NP, effectively addressed inflammatory reactions and maintained ECM anabolic and catabolic balance in a rat model.^56^ Pan et al. created a thermo‐sensitive HA hydrogel capable of controlled gefitinib release, which promotes ECM production and activates autophagy markers for treating IVDD.^57^ Additionally, Chen et al. developed a siRNA delivery system by combining hyaluronic aldehyde (HA‐CHO) with polyamide amine/siRNA complexes, achieving sustained silencing of STING expression, promoting tissue regeneration, and alleviating IVDD through inflammation reduction.^58^ Lastly, Zhao et al. utilized an extrusion‐crushing method to prepare a HA particulate hydrogel embedded with ZrO₂ nanoparticles for X‐ray and CT visualization. This hydrogel restored normal IVD mechanics in a rabbit degenerative disc model while preserving disc structural integrity.^59^

Many researchers have incorporated stem cells into HA hydrogels to create a more supportive growth environment. Hu et al. encapsulated bone marrow mesenchymal stem cells and salvianolic acid B in HA hydrogels and found that salvianolic acid B effectively reduces the apoptosis of bone marrow mesenchymal stem cells (BMSCs) by activating the JAK2‐STAT3 pathway.^60^ Similarly, Choi et al. developed a HA‐methylcellulose hydrogel loaded with mesenchymal stem cells. Their findings indicated that this hydrogel was more effective in promoting IVDD repair compared to simple cell injection.^61^ Furthermore, using a light‐crosslinked hydrogel made of HA and collagen to load bone marrow mesenchymal stem cells is an innovative approach. This hydrogel uses riboflavin as a crosslinking agent and gels rapidly under blue light within 3 min. Incorporating the FG4592 growth factor into the hydrogel enhances ECM production and promotes the proliferation and differentiation of bone marrow mesenchymal stem cells.^62^ In addition to mesenchymal stem cells, Wang et al. identified a new type of fibrocartilage‐like AF cell with stem cell properties using single‐cell RNA sequencing. They then loaded these novel cells onto silk fibroin and HA hydrogels, adding fibrocartilage cell‐inducing supplements, and successfully observed the reconstruction of AF defects.^63^

Microfluidics is a technology for controlling and manipulating fluids on a microscale, which has seen increasing attention in recent years for its applications in hydrogel microsphere synthesis. Chang et al. employed microfluidic technology to create HA methacrylate (HAMA) microspheres, which facilitated effective loading and stable release of liposomes, making them ideal carriers for gene drugs.^64^ Furthermore, Shen et al. used microfluidics to develop HAMA microspheres designed for localized lactic acid removal. These microspheres are capable of mitigating oxidative and inflammatory stress, promoting ECM production, and restoring disc height. By targeting lactic acid accumulation in the local microenvironment, these nanoscale enzymatic microspheres significantly enhance the regeneration of ischemic tissue and improve the repair of IVD.^65^

#### 6.1.2 Collagen/gelatin

Collagen is a structural protein primarily composed of amino acid chains, including glycine, proline, and others. It is widely distributed in the connective tissues of both humans and animals, playing a crucial role in maintaining tissue structure, elasticity, and strength. The triple‐helix structure of collagen provides it with exceptional stability and resilience. These characteristics make collagen an ideal material for synthesizing biological scaffolds.^66^, ^67^ Takeoka et al. successfully developed a novel low‐adhesive scaffold collagen (LASCol) using actinidin hydrolysis technology. Compared to traditional atelocollagen, LASCol exhibits enhanced solubility and faster degradation while significantly reducing antigenicity. Notably, LASCol can effectively induce cells to form stable three‐dimensional spherical structures. Additionally, LASCol excels in promoting the formation of ECM, which helps delay the histological degeneration of IVD.^68^

Collagen is prized for its exceptional structural support and strength, while HA is celebrated for its hydration and flexibility. Consequently, these two components are frequently combined in hydrogel design. Sloan Jr. et al. introduced an innovative method by using HA injections to enhance the NP and combining this with photo cross‐linked collagen patches to repair the AF. This approach is designed to prevent degeneration and maintain mechanical function in sheep lumbar spines after discectomy. Not only does this combined method effectively restore NP hydration and repair AF defects, but it also preserves the original torsional and compressive stiffness for up to 6 weeks post‐injury.^69^ Furthermore, Koo et al. developed a shape‐memory collagen‐HA hydrogel that significantly reduces mechanical allodynia in the rat tail, maintains high water content in the IVD, and supports disc structure by restoring ECM proteins.^70^ Bowles et al. developed a hydrogel nucleus pulposus and a collagenous annulus fibrosus, which, when implanted into the rat caudal spine, maintained disc height, produced new extracellular matrix, and integrated with the native spine to form a functional motion segment with mechanical properties similar to native intervertebral discs.^71^

Gelatin is derived from collagen through a hydrolysis process that breaks down collagen's triple‐helix structure. It dissolves in water when heated and forms a gel‐like substance upon cooling.^72^, ^73^ Some existing gelatin hydrogels have self‐healing properties, enabling them to restore their original structure and function after damage, thereby improving the material's reliability and stability. Tian et al. developed a self‐healing gelatin hydrogel loaded with kartogenin, which allows for localized and sustained release of kartogenin. This hydrogel promotes ECM synthesis in vitro and helps mitigate the progression of IVDD in vivo by restoring redox balance.^74^ Similarly, Hu et al. created a self‐healing hydrogel made from thiolated gelatin and β‐cyclodextrin, incorporating melatonin to promote AF regeneration. Their results showed that this hydrogel supports in situ regeneration of AF tissue, thereby preventing disc degeneration by maintaining NP hydration.^75^ To better replicate the mechanical environment of the IVD, Yang et al. developed a novel injectable self‐healing hydrogel with dual dynamic cross‐links (Prussian blue nanoparticles@oxidized HA/borax/gelatin). This hydrogel exhibits antibacterial and antioxidant properties, along with great mechanical performance, and is capable of reversing the disordered microenvironment of IVDD.^76^ Wang et al. used viscoelastic and thixotropic colloidal gels, which simulate the biomechanical microenvironment of IVD, as injectable scaffolds for delivering mesenchymal stem cells. This gelatin hydrogel demonstrates good biocompatibility and biodegradability, supporting the differentiation of BMSCs into NP‐like cells. Following injection, the hydrogel exhibits outstanding leak‐proof performance, enhanced cell survival, and effective regeneration of degenerative NP.^77^

The functionality of gelatin hydrogels can be greatly enhanced by incorporating specific biological factors or small molecule nucleic acids during their preparation. Yahia et al. introduced a combination of platelet‐rich plasma (PRP) and simvastatin encapsulated in polymeric nanomicrocages into gelatin‐based hydrogels. These hydrogels exhibited a swelling ratio exceeding 500% after 24 h, with a drug release rate of up to 88.4% after 21 days. The inclusion of PRP improves tissue healing and reduces inflammation.^78^ Furthermore, tetrazine‐norbornene bioorthogonal ligation was combined with gelatin to develop an injectable bioorthogonal hydrogel. This hydrogel significantly enhances tissue repair, including tissue structure and matrix synthesis, and supports functional recovery by improving water retention and alleviating pain through the promotion of matrix synthesis, facilitated by the encapsulation of transforming growth factor‐β (TGF‐β).^79^ Chen et al. developed a self‐healing, adhesive, and antibacterial multifunctional gelatin hydrogel using multiple dynamic bonds. This hydrogel can continuously release siRNA to target the P65/NLRP3 inflammatory signaling pathway, thereby suppressing cellular inflammation and significantly enhancing IVD regeneration.^80^

#### 6.1.3 Alginate

Alginate, a natural polysaccharide extracted from brown algae and primarily found in their cell walls, boasts great biocompatibility, customizable physical properties, and ease of processing. These attributes make alginate hydrogel highly promising for diverse applications.^81^ For instance, Wang et al. developed a “mito‐engine” system and incorporated it into an alginate/gelatin hydrogel responsive to reactive oxygen species (ROS). This system enhances cellular mitochondrial membrane potential, regulates ROS levels, prevents oxidative damage, and ultimately promotes the regeneration of degenerated IVD.^82^

**FIGURE 4:**
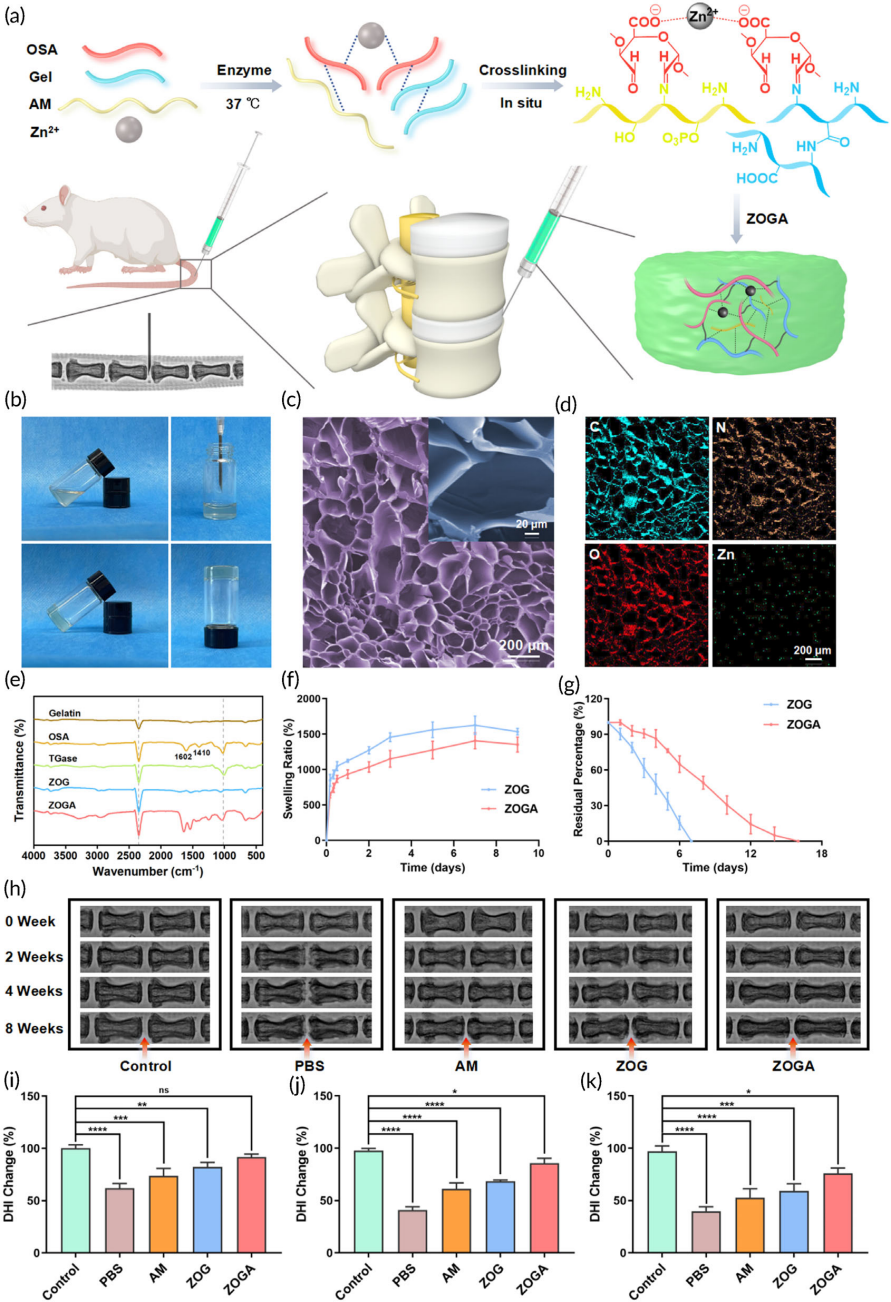
High‐strength multifunctional biohydrogel for ameliorating intervertebral disc degeneration. (a) Preparation and transplantation of ZOGA. (b) Thermosensitive gelation: liquid precursor (top) versus formed hydrogel after 37°C incubation for 6 h (bottom). (c) Representative SEM image of ZOGA illustrating its porous microstructure. (d) Elemental mapping images of ZOGA. (e) ATR‐FTIR analysis of the chemical structures of ZOGA. (f) The hygroscopicity of ZOG and ZOGA. (g) The degradability of ZOG and ZOGA. (h) X‐ray images of rat coccygeal vertebrae before surgery, and at 2, 4, and 8 weeks after surgery. (i–k) DHI changes in different groups at 2, 4, and 8 weeks after surgery (AM, AntagomiR‐204‐3p; DHI, disc height index; Gel, gelatin; PBS, phosphate buffered saline; OSA, oxidized sodium alginate; ZOG, zinc‐OSA‐gelatin; ZOGA, zinc‐OSA gelatin‐AM; ATR‐FTIR, attenuated total reflectance Fourier transform infrared spectroscopy).^84^ Copyright, 2023 Elsevier.

Given the tunable physical properties of alginate hydrogels, Zamboni et al. incorporated curcumin/polylactic acid nanoparticles into an alginate–gelatin hydrogel. This not only enhanced the compressive strength and mechanical performance of the hydrogel but also maintained its original swelling and degradation characteristics. Furthermore, the hydrogel can inhibit immune cell activation and inflammation, demonstrating significant potential in tissue engineering.^83^ Similarly, Chen et al. designed a high‐strength multifunctional nucleic acid delivery platform: zinc‐oxidized alginate–gelatin hydrogel. This hydrogel exhibits great moisture absorption, antibacterial properties, biocompatibility, and biodegradability, and can form a high‐strength collagen network through crosslinking with NP tissue, significantly improving the mechanical performance of IVD (Figure 4).^84^ Additionally, melatonin/mesoporous bio‐glass/alginate hydrogel is a biomaterial combining mechanical performance and drug release functionality, with a compressive load range of 0.75–2.75 MPa, matching the natural IVD's compressive load range of 0.1–0.9 MPa. Moreover, this hydrogel provides sustained release of melatonin, which alleviates IL‐1β‐induced oxidative stress and reduces inflammation associated with IVDD.^85^

Given the great biocompatibility of alginate, many researchers have co‐cultured cells with alginate hydrogels to investigate the impact of this biomaterial on cell function. Ukeba et al. embedded BMSCs and NPCs in ultra‐pure alginate hydrogels to evaluate their regenerative potential for degenerative IVD. The results demonstrated that, under three‐dimensional co‐culture conditions, the gene expression levels of NPC markers, growth factors, and ECM components were significantly higher compared to single‐cell culture.^86^ Additionally, other studies have encapsulated stem cells in alginate and gelatin microgels, revealing that these microgels can inhibit pyroptosis activation by maintaining mitochondrial homeostasis while promoting the migration, proliferation, and differentiation of mesenchymal stem cells (MSCs).^87^ In addition to MSCs, NPCs are also frequently co‐cultured with alginate hydrogels. Lekerika et al. utilized alginate as a three‐dimensional culture material mixed with primary human NPCs. Their results showed that the number of cells in the core region of the hydrogel was significantly higher than in the surface region, indicating the great biocompatibility of alginate.^88^ Furthermore, Tan et al. have demonstrated that, compared to plain alginate hydrogels, alginate hydrogels supplemented with integrin and syndecan produce NPCs with enhanced cell viability, biosynthetic activity, and NP‐specific protein expression.^89^ Additionally, a “self‐ contained” sodium alginate hydrogel carrying NPCs has been designed to modulate the local inflammatory microenvironment. Ca^2+^ and Mg^2+^ are released from the silicate ceramics within the hydrogel, with Ca^2+^ acting as a crosslinking agent for the alginate, while Mg^2+^ facilitates local immune modulation. Ultimately, this hydrogel promotes the proliferation of NPCs and the regeneration of NP tissue, effectively delaying IVDD.^90^

#### 6.1.4 Chitosan

Chitosan is a natural polymer derived from the shells of crustaceans such as shrimp and crabs, and it is renowned for its great properties, including biocompatibility, biodegradability, and antibacterial activity. Researchers have developed various chitosan‐based hydrogels as delivery systems for cells and drugs, highlighting their substantial potential in drug delivery, tissue engineering, and other medical applications.^91^, ^92^ Wang et al. designed a manganese oxide‐functionalized thermosensitive chitosan hydrogel for the transplantation of bone marrow mesenchymal stem cells to treat IVDD. This hydrogel improves the ROS environment, protects the bone marrow mesenchymal stem cells from mechanical stress, and promotes their differentiation into NP‐like cells.^93^ Similarly, a thermosensitive chitosan‐based hydrogel functionalized with celecoxib has been demonstrated to effectively fill local tissue defects in IVD and maintain spinal stability. This hydrogel provides a controlled release of celecoxib, thereby maximizing its anti‐inflammatory effects and delaying post‐operative disc degeneration.^94^ Celecoxib, a commonly used non‐steroidal anti‐inflammatory drug in clinical practice, is frequently employed in tissue engineering. Nezadi et al. incorporated celecoxib into an injectable, self‐healing, shear‐thinning mixed hydrogel. This hydrogel simulates the swelling and mechanical rheological behavior of natural human NP and controls the release of celecoxib over a period of 2 weeks. Furthermore, this hydrogel system exhibits good cavity‐filling capabilities and enhances ECM secretion, indicating promising potential for the treatment of disc degeneration.^95^

Hydrogels represent macroscopic delivery systems; microspheres are considered microscopic delivery systems, and nanoparticles are classified as nanoscopic delivery systems. The integration of multi‐scale delivery systems is an innovative approach in drug delivery technology, aiming to achieve efficient, precise, and controlled release of drugs across various scales. This approach leverages the benefits of multiple scales to enhance drug targeting, bioavailability, and therapeutic efficacy.^96^, ^97^ Zhao et al. developed an injectable and biodegradable nanocomposite hydrogel by cross‐linking chitosan and HA and incorporating hydroxyapatite‐epigallocatechin‐3‐gallate nanorods. This multi‐scale delivery system not only offers the structural support of hydrogels but also allows for the controlled release of nanorods. Biologically, this hydrogel regulates the balance between ECM synthesis and degradation and promotes the differentiation of macrophages into M2 anti‐inflammatory cells, thereby creating a conducive ECM environment.^98^ Wang et al. created an in situ cross‐linked multi‐scale delivery system by embedding tannic acid nanoparticles into chitosan hydrogels. This system boasts great biocompatibility, degradability, pH responsiveness, adjustable gelation time, and mechanical strength. Biologically, it can inhibit inflammation and regulate ECM metabolic balance.^99^ Furthermore, Ma et al. combined growth differentiation factor 5 (GDF5)‐loaded poly(lactic‐co‐glycolic acid) (PLGA) microspheres with a mixture of decellularized NP matrix and chitosan hydrogel, achieving the integration of microscopic and macroscopic delivery systems. When co‐cultured with NP stem cells, this hydrogel efficiently, precisely, and slowly releases GDF5, facilitates the differentiation of NP stem cells into NP‐like cells, and supports the regeneration of NP tissue, highlighting the promising application potential of multi‐scale delivery systems.^100^

#### 6.1.5 Polypeptide

Peptides are organic compounds composed of amino acids linked by peptide bonds, with chain lengths ranging from two to several dozen amino acids. The formation of peptide hydrogels generally depends on intermolecular forces such as hydrogen bonds, hydrophobic interactions, and ionic bonds. These forces enable peptide molecules to self‐assemble into a stable three‐dimensional network structure, which traps water and forms a gel.^101^, ^102^ The resulting peptide hydrogels can be utilized as biomaterials for cell culture or as carriers in drug delivery systems, allowing for precise control over drug release rate and location.^103^

Many researchers have investigated the integration of NPCs into peptide hydrogels to examine their biological properties. Bian et al. employed microfluidic technology to create peptide–cell‐hydrogel microspheres that were covalently grafted with APETx2 and loaded with NPCs. These innovative microspheres significantly not only reduce inflammatory cytokine storms over an extended period but also enhance the proliferation of NPCs and increase the deposition of ECM. Such effects contribute to mitigating IVDD and fostering its regeneration, offering an effective approach for tissue regeneration under excessive inflammatory conditions.^104^ Additionally, a peptide‐functionalized PEG hydrogel scaffold was designed for NP cell encapsulation. Both in vitro and in vivo culture results confirmed that this system serves as an effective cell carrier, promoting cell retention within the IVD, enhancing NP cell vitality, increasing biosynthetic activity, and facilitating matrix deposition.^105^ Ligorio et al. used acidic (pH = 4) and basic (pH = 9) octapeptide FEFKFEFK hydrogels to investigate the impact of non‐physiological pH on encapsulated NPCs. While the initial pH of the scaffold did not significantly affect cell encapsulation, the presence of graphene oxide reduced inflammation levels and boosted matrix protein production, thereby creating a more favorable microenvironment for NP cell growth.^106^ Another functionalized peptide, RAD/SA1, has the ability to self‐assemble into a three‐dimensional (3D) nanofiber hydrogel scaffold in acidic environments. This scaffold facilitates the proliferation of human NP mesenchymal stem cells and enhances their secretion of ECM components.^107^

In addition to serving as vehicles for cell delivery, peptide hydrogels are frequently utilized for the transport of extracellular vesicles (EVs) or biological factors. Peng et al. have crafted a decellularized tissue matrix (DTM) hydrogel, formed from an arginine–glycine–aspartic acid tripeptide complex, specifically designed for extracellular vesicles delivery. This hydrogel targets the HIPK2/p53 pathway, thereby augmenting the bioavailability of EVs and counteracting cellular aging, ultimately easing IVDD.^108^ Meanwhile, in the realm of GDF‐5 delivery, Conley et al. have introduced a dynamic and multifunctional nano‐hybrid peptide hydrogel. This hydrogel boasts not just injectability, biocompatibility, and biodegradability, but also exhibits therapeutic properties. It catalyzes the elimination of ROS and facilitates ECM remodeling. Through the consistent delivery of GDF‐5, the hydrogel proficiently curtails immune responses and revitalizes the regenerative microenvironment of the ECM.^109^

#### 6.1.6 Fibrin

Fibrin is a protein formed from fibrinogen during blood clotting. Fibrin‐based hydrogels, with their network structure, allow for the control of mechanical properties such as elasticity and strength by adjusting the concentration of fibrin and crosslinking conditions. Hydrogels with optimal mechanical properties can effectively prevent recurrent disc herniation after discectomy.^110^, ^111^ Long et al. employed a composite repair strategy for annulus fibrosus repair. They used a fibrin–genipin (FibGen) hydrogel adhesive, poly(trimethylene carbonate) (PTMC) scaffolds, and polyurethane membranes. The FibGen adhesive was used to seal the annulus fibrosus defect; the PTMC scaffolds provided a space‐filling solution and enabled cell delivery; and the polyurethane membranes acted as a barrier to prevent herniation of intervertebral disc tissue. This composite approach aimed to restore the biomechanics of the intervertebral disc and minimize the risk of herniation.^112^ Panebianco et al. proposed an innovative composite strategy by incorporating oxidized alginate microspheres into high‐modulus fibrin hydrogels. This approach is designed to balance biomechanics and biological performance in the complex environment of the IVD. The composite hydrogel not only reduces cell apoptosis and preserves phenotype gene expression but also delivers both biomechanical and biological repair effects, thereby decreasing the risk of postoperative disc herniation.^113^ Additionally, DiStefano et al. developed an injectable interpenetrating network hydrogel made from fibrin linked with fibronectin and poly(ethylene glycol) diacrylate. This hydrogel network effectively seals post‐surgical annulus defects and prevents recurrent disc herniation.^114^

Fibrin hydrogels incorporating bioactive factors preserve the mechanical properties of the fibrin network while allowing for the controlled release of these factors to achieve specific biological functions. For instance, the study by Likhitpanichkul et al. investigated the use of FibGen as a drug delivery system for the anti‐TNFα drug, infliximab, in the treatment of intervertebral disc degeneration. The results showed that FibGen could sustain the release of infliximab and reduce the production of pro‐inflammatory cytokines by annulus fibrosus cells.^115^ Li et al. integrated silk fibers into sodium alginate hydrogels to enhance their mechanical strength and added PRP to boost the bioactivity of the hydrogels. The results indicate that the hydrogel can withstand stresses up to 50 kPa with 30% strain, showcasing outstanding mechanical properties and leakage resistance. Moreover, this nanofiber‐enhanced hydrogel can gradually release PRP over 40 days, facilitating cell proliferation and tissue microenvironment reconstruction.^116^ Gao et al. incorporated quercetin into fibrin to develop a fibrin delivery system with good biocompatibility and sustained release characteristics. This system effectively reduces inflammation associated with IVDD and regulates matrix synthesis and degradation.^117^ Additionally, Wang et al. embedded platelet‐derived vesicles into a fibrin matrix to develop a hydrogel with biocompatibility, biodegradability, and sustained release properties. This hydrogel can prevent cell pyroptosis by correcting abnormal fatty acid metabolism, while also mitigating inflammation and maintaining ECM homeostasis.^118^

#### 6.1.7 Chondroitin sulfate

Chondroitin sulfate is a naturally occurring compound predominantly found in articular cartilage and other connective tissues. It plays a crucial role in maintaining joint lubrication, reducing inflammation, and promoting tissue repair.^119^, ^120^ Often, chondroitin sulfate is cross‐linked with other natural substances such as collagen and gelatin to create multifunctional biological hydrogels. For instance, Zhou et al. developed a type II collagen/chondroitin sulfate composite hydrogel delivery system for adipose‐derived stem cells (ADSCs). This system demonstrates great biocompatibility and enhances the expression of disc‐specific genes. In vivo injection of this hydrogel resulted in partial restoration of IVD height, water content, and structure.^121^ Beyond its combination with collagen, chondroitin sulfate is also frequently cross‐linked with gelatin. Liu et al., for example, designed a chondroitin sulfate/gelatin hydrogel for the delivery of Glutaredoxin3 and EVs. This hydrogel is injectable, biodegradable, and responsive to ROS, making it effective in delivering EVs‐Glutaredoxin3 to regulate redox balance, reduce mitochondrial damage, and mitigate NP cell aging, thereby delaying IVDD.^122^ Additionally, Borrelli et al. combined the DTM from bovine NP tissue with functionalized chondroitin sulfate to create an injectable self‐assembling biomimetic material. The chondroitin sulfate in this material supports the synthesis of the ECM and helps maintain cell morphology during three‐dimensional culture.^123^

### 6.2 Synthetic and semi‐synthetic hydrogels

#### 6.2.1 Gelatin methacrylate

Gelatin methacrylamide (GelMA) is a hydrogel material derived from natural gelatin and chemically modified to enhance its performance. Key features include great biocompatibility, tunable physical properties, and photo‐crosslinking capabilities, which make it highly suitable for applications in tissue engineering and regenerative medicine.^124^ GelMA is frequently combined with various drugs, bioactive proteins, cells, and nanoparticles to create composite materials that effectively exhibit anti‐inflammatory, antioxidant, cell regeneration‐promoting, and tissue repair functions.^125^

**FIGURE 5:**
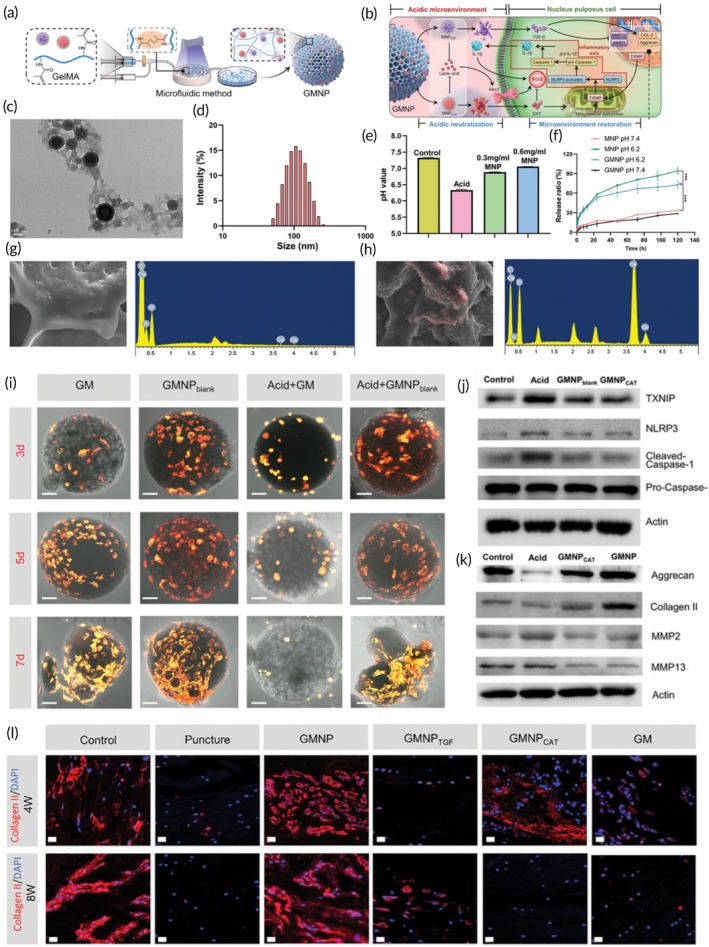
Hydrogen ion capturing hydrogel microspheres with inflammation‐reversing effects for improving intervertebral disc degeneration. (a) Preparation of GMNP loaded with MNP via the microfluidic device. (b) The effect of GMNP on stabilizing the microenvironment via capturing hydrogen ion, blocking NLRP3 inflammasome cascade axis, and enhancing extracellular matrix synthesis in intervertebral disc degeneration. (c) Transmission electron microscope image of MNP. Scale bar: 50 nm. (d) Size distribution of MNP by DLS analysis. (e) Evaluation of the acidic neutralization ability of MNP using 0.3 or 0.6 mg/mL MNP. (f) Drug release profiles of MNP and GMNP under neutral or acidic conditions. (g, h) Surface elemental analysis of GM (g) and GMNP (h). (i) Confocal images of different formulations co‐cultured with NPCs under different conditions. Scale bar: 50 μm. (j) WB bands of NLRP3 inflammasome‐related protein in NPCs, including TXNIP, NLRP3, cleaved‐caspase‐1, pro‐caspase‐1, and 𝛽‐actin. (k) WB bands of regeneration‐related protein in NPCs, including Aggrecan, Collagen II, MMP2, MMP13, and 𝛽‐actin. (l) Collagen II immunofluorescent staining of rat intervertebral discs. Scale bar: 50 μm (Cat, catalase; GelMA, gelatin methacrylamide; GM, Gelma microspheres; GMNP, hydrogen ion capturing hydrogel microsphere; MNP, mineralized nanoparticle; MMP, matrix metalloproteinases; NPCs, nucleus pulposus cells; NLRP3, nod‐like receptor thermal protein domain associated protein 3; TGF‐β, transforming growth factor‐β; TXNIP, thioredoxin interacting protein).^127^ Copyright, 2024 Wiley.

In terms of anti‐inflammatory effects, Zhu et al. developed multifunctional GelMA microspheres incorporating vanillin for the localized delivery of TGFβ3. These microspheres not only enhanced the release kinetics of TGFβ3 but also effectively suppressed the inflammatory response in NPCs and promoted ECM secretion. Upon injection into the body, these microspheres can reduce inflammation and oxidative stress, maintain the water content of the NP and the height of the IVD, and preserve the integrity of the IVD structure.^126^ Similarly, Zheng et al. designed mineralized GelMA microspheres with hydrogen ion‐capturing capabilities through biomimetic mineralization and microfluidic technology to deliver TGFβ. These microspheres neutralize the acidic microenvironment by capturing excess hydrogen ions via a calcium carbonate mineralization layer and release TGF‐β and catalase to counteract both endogenous and exogenous inflammatory stimuli. In vivo, these hydrogel microspheres establish a continuously stable ecological niche that suppresses inflammation and supports the regeneration of degenerated IVD (Figure 5).^127^

**FIGURE 6:**
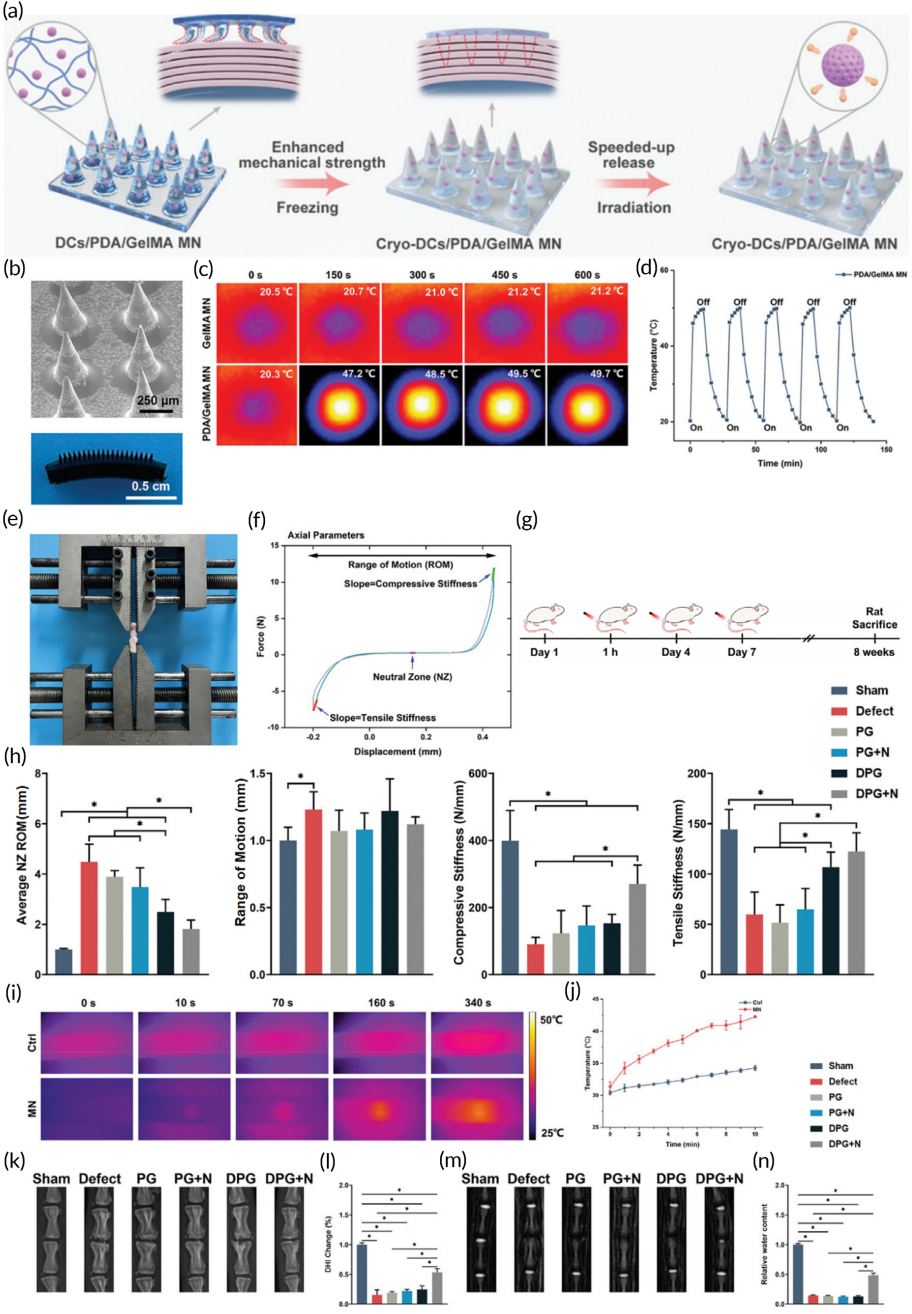
High‐strength smart microneedles with “offensive and defensive” effects for intervertebral disc repair. (a) The preparation of the composite microneedles. The mechanical strength of the microneedles is improved by the freezing treatment to enable it to penetrate the annulus fibrosus of the intervertebral disc, and achieve remote control of sped‐up release of the drug and hyperthermia by the near infrared. (b) Macroscopic and SEM representative images of the MNs. (c, d) Photothermal image and photothermal stability of MNs. (e) Axial tension–compression biomechanical testing on rat caudal vertebra. (f) Representative force–displacement curve of Sham group. (g) Experimental timeline. Rats received MNs treatment post‐surgery. NIR treatment began 1 h later and repeated every 3 days. At 8 weeks, biomechanical testing was performed, followed by rat sacrifice. (h) Results of torsional neutral zone length, range of motion, compressive stiffness and tensile stiffness. (i) The photothermal images of MNs under 808 nm NIR irradiation in rat caudal vertebra. (j) The temperature changes of MNs. (k) X‐ray images of rat caudal vertebra at week 8. (l) Quantitative analysis of DHI. (m) MRI images of rat caudal vertebra at week 8. (n) Quantitative analysis of the relative water content of intervertebral disc (DCs, diclofenac sodium; DHI, disc height index; DPG, DCs/PDA/GelMA MN; DPG+N, DCs/PDA/GelMA MN+NIR; GelMA, gelatin methacrylamide; MRI, magnetic resonance imaging; MN, microneedle; NZ, neutral zone; NIR, near infrared; PDA, polydopamine; PG, PDA/GelMA MN; PG+N, PDA/GelMA MN+NIR; ROM, range of motion).^129^ Copyright, 2024 Wiley.

In addition to TGFβ delivery, some researchers have explored the use of clinically common non‐steroidal anti‐inflammatory drugs (NSAIDs) in anti‐inflammatory materials. For instance, Liu et al. incorporated aspirin‐loaded liposomes into photo‐crosslinked GelMA hydrogels. Their study demonstrated that aspirin could be controllably released from the hydrogel, effectively covering the inflammatory period following IVD surgery and inhibiting the expression of inflammatory factors.^128^ Sodium diclofenac, another commonly used NSAID, was utilized by Meng et al. who developed intelligent polydopamine/GelMA microneedles (a cross‐linked hydrogel‐based drug delivery system). These microneedles, which can penetrate the AF tissue of the IVD via a minimally invasive approach, achieve remote‐controlled accelerated release and thermal therapy of sodium diclofenac through near‐infrared stimulation. These intelligent microneedles can “target” the inflammatory microenvironment extracellularly, reduce cellular damage, and elevate heat shock protein levels inside cells to enhance their defense against hostile conditions, thus achieving a combined “attack and defense” effect (Figure 6).^129^ Lastly, epigallocatechin‐3‐gallate, a natural anti‐inflammatory substance, was conjugated to boronic acid‐modified GelMA to form an anti‐inflammatory and antioxidant hydrogel. This hydrogel can responsively release drugs under high levels of ROS and acidic conditions, providing cellular protection.^130^

In terms of antioxidant properties, natural antioxidants can be embedded in biomaterials to mitigate oxidative stress‐induced cellular damage. Li et al. developed a novel fucoidan‐functionalized GelMA microsphere that gradually releases fucoidan to induce antioxidant enzymes in response to oxidative stress. Furthermore, after in situ injection, the GelMA microsphere effectively preserves ECM components and maintains hydration in the NP tissue.^131^ Luteolin, a natural compound widely found in plants, exhibits potent antioxidant properties. Liu et al. created an injectable, drug‐loaded, dual‐stimuli‐responsive adhesive hydrogel‐GelMA/HA/luteolin to regulate the microenvironment of IVDD. This hydrogel features an extended enzymatic degradation time and continuously releases luteolin, demonstrating exceptional free radical scavenging efficiency and strong antimicrobial activity. In situ injection of this hydrogel effectively maintains disc hydration, restores disc height, and promotes NP regeneration.^132^ Additionally, Xu et al. prepared MnO_2_/GelMA composite hydrogels with good biocompatibility and a porous structure, which supports cell proliferation. The incorporation of MnO_2_ nanoparticles further enhances antioxidant properties and induces cell autophagy via the SIRT1/NRF2 pathway, thus preventing oxidative damage under oxidative stress.^133^

Anti‐inflammatory and antioxidant mechanisms are somewhat intertwined, and these two processes are closely related in biological functions, influencing each other through various pathways. Consequently, materials with anti‐inflammatory effects often also exhibit certain antioxidant properties. Li et al. grafted chitosan nanoparticles loaded with highly reducible black phosphorus quantum dots onto GelMA microspheres to create an engineered hydrogel microsphere with balanced oxygen metabolism. This microsphere maintains equilibrium between ECM synthesis and degradation by regulating the positive feedback between imbalanced oxygen metabolism and inflammation in IVD.^134^ Similarly, Tang et al. combined GelMA, HA, histidine, and Mg^2+^ to develop a charged hydrogel microsphere system. This system retains the activity of loaded nanoparticles in the degenerative disc microenvironment through Mg^2+^ charges in the microspheres, effectively supporting the nanoparticles in combating ROS‐induced inflammation and aging.^135^

#### 6.2.2 Polyethylene glycol

Polyethylene glycol (PEG), a linear polymer consisting of ethylene glycol units linked by ether bonds, serves as a great foundation for hydrogels. By manipulating the molecular weight and concentration of PEG, one can precisely regulate the hydrogels' water absorption capacity and mechanical attributes. Furthermore, PEG acts as a crosslinking agent, interacting with various monomers to establish the intricate network structure inherent to hydrogels.^136^, ^137^ Huang et al. have innovated a chitosan/polyethylene glycol injectable hydrogel that forms rapidly in situ. This hydrogel undergoes swift gelation through a Schiff base reaction between chitosan and PEG. Remarkably, under blue light exposure, the hydrogel's strength experiences a notable surge within mere seconds. The hydrogel demonstrates minimal cytotoxicity in laboratory settings and when cocultured with cells. Moreover, when implanted in a living organism, it effectively mitigates the progression of IVDD by creating a physical barrier.^138^

Due to its great physicochemical properties and biocompatibility, PEG hydrogel holds significant potential for delivering biological agents such as nucleic acids, drugs, and proteins. Feng et al. developed a sustained, bioresponsive two‐stage miRNA delivery system aimed at inhibiting fibrosis and thereby delaying IVDD. In the first stage, increased MMP levels in the localized fibrotic area trigger the degradation of the hydrogel, resulting in the continuous release of polymeric micelles within the affected tissue. In the second stage, the responsive detachment of the PEG shell from the micelles, prompted by MMPs, facilitates the effective cellular uptake of miR‐29 and its escape from endosomes.^139^ Similarly, Chen et al. developed a multifunctional PEG hydrogel with injectable, self‐healing, antimicrobial, and biodegradable properties by coordinating four‐armed PEG‐SH with silver ion solutions. This hydrogel delivers the loaded agomir874 to degenerative discs, establishing an in situ gene‐hydrogel microenvironment that regulates the balance of ECM synthesis and degradation, thus promoting tissue regeneration.^140^

Beyond nucleic acid delivery, PEG hydrogels are also employed in drug delivery applications. Chen et al. engineered a thermosensitive PLGA–PEG–PLGA hydrogel loaded with bevacizumab, which provides a controlled release of the drug to inhibit vascular endothelial growth factor expression and thereby enhance ECM synthesis.^141^ Additionally, Zheng et al. combined PEG and PLGA to design a thermosensitive, ROS‐responsive hydrogel containing a synthetic growth hormone‐releasing hormone analog (MR409). This hydrogel facilitates the localized release of MR409, inhibiting ROS‐induced secretory autophagy and IL‐1β secretion, thereby mitigating IVDD.^142^ PRP is a biological preparation derived from autologous blood, rich in growth factors that support tissue healing and recovery. It is widely used in clinical settings for managing joint pain and restoring function. Choi et al. utilized microfluidic technology to create biodegradable PEG microspheres loaded with PRP. By encapsulating and releasing PRP, this PEG delivery system promotes tissue regeneration and wound healing.^143^

#### 6.2.3 Polycaprolactone

Polycaprolactone (PCL) is a biodegradable polymer produced via the ring‐opening polymerization of caprolactone. It is highly valued for its exceptional biocompatibility and flexibility. Due to its superior swelling properties, high drug loading capacity, and controlled drug release characteristics, PCL hydrogels are frequently utilized as drug delivery systems.^144^, ^145^ Wang et al. used electrospinning technology to create PCL nanofibers loaded with low doses of celecoxib. These nanofibers can gradually release celecoxib, effectively providing anti‐inflammatory effects and promoting ECM synthesis and metabolism.^146^ Beyond its role as a drug carrier, PCL also shows promise as a supportive scaffold. Zheng et al. employed an innovative coaxial electrospinning method to combine PCL with chitosan, creating a core–shell structure scaffold infused with perfluorotributylamine. This scaffold is capable of continuously releasing oxygen for up to 144 h, thereby protecting AF stem cells from hypoxia‐induced apoptosis and enhancing cell proliferation, migration, and ECM generation.^147^

The mechanical properties of PCL can be finely tuned by adjusting processing conditions or by blending it with other materials. This flexibility makes PCL ideal for applications requiring specific mechanical strength and elastic modulus, such as designing artificial AF. Gullbrand et al. used HA to prepare an artificial NP, PCL to create an artificial AF and CEP, and developed a tissue‐engineered, endplate‐modified disc‐like angle‐ply structure. This artificial IVD achieved functional integration under physiological loads and demonstrated mechanical compressive properties comparable to natural discs after 8 weeks of implantation in goat cervical IVD, suggesting its potential for clinical application.^148^ Similarly, the artificial IVD designed by Kim et al. also features a PCL‐based biomimetic AF and HA‐based biomimetic NP. After 5 weeks of implantation in rat tail vertebrae, the artificial disc maintained its structural integrity.^149^ Additionally, Ashinsky et al. combined PCL with polyethylene oxide to create a tissue‐engineered disc‐like angle‐ply structure, facilitating cell infiltration and uniform distribution of the matrix.^150^ Gluais et al. developed a cell‐free PCL fiber scaffold that mimics the natural AF in both morphology and mechanical properties, effectively promoting spontaneous cell colonization and proliferation. When implanted into a sheep AF injury model, the collagen fibers in each layer of the scaffold were uniformly aligned and seamlessly integrated with the surrounding AF tissue.^151^

#### 6.2.4 Polylactic acid/polyglycolic acid

Polylactic acid (PLLA), produced through the polymerization of lactic acid, is a biodegradable material known for its great mechanical properties and is widely used in packaging and medical applications.^152^ Han et al. employed electrospinning technology to create PLLA scaffolds loaded with ibuprofen and HA scaffolds loaded with TGFβ3. The rapid release of ibuprofen improves the inflammatory microenvironment, while the sustained release of TGFβ3 promotes the formation of new ECM. These dual‐drug delivery scaffolds are assembled into a stratum corneum‐like structure, forming a highly biomimetic fiber ring that can be implanted into IVD defects or used for disc replacement.^153^ Nakielski et al. assessed the safety and feasibility of chitosan and chondroitin sulfate‐modified electrospun nanofiber micro‐scaffolds (PLLA/PLGA) for treating degenerative NP tissue in large animal models. The results showed that these scaffolds were non‐toxic, promoted cell adhesion, and effectively delivered cells into the high‐pressure environment of the IVD.^154^

PLGA, synthesized through the copolymerization of lactic acid and glycolic acid, is also biodegradable with controllable degradation rates and good biocompatibility.^155^ It is extensively used in drug delivery and tissue engineering. Wang et al. developed a cartilage‐derived matrix@PLGA‐GelMA/PRP composite hydrogel as a carrier for ADSCs‐based IVD repair therapy. This composite hydrogel releases KGN, which stimulates ADSCs to differentiate into NP‐like phenotypes and enhances their antioxidant capacity by activating the Nrf2/TXNIP/NLRP3 axis, showcasing the promising potential of stem cell therapy for IVDD.^156^

#### 6.2.5 Polyvinyl alcohol

**FIGURE 7:**
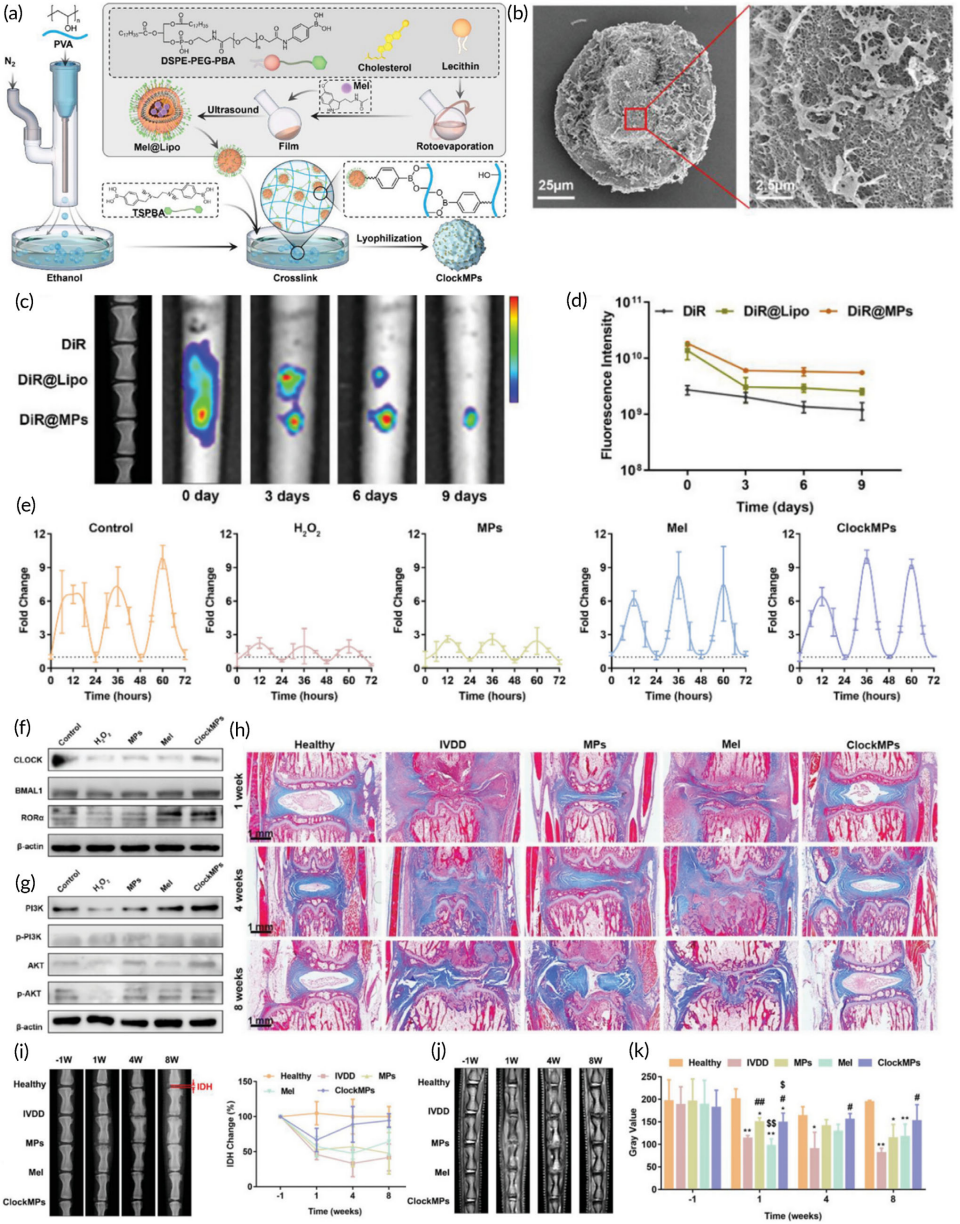
Circadian clock‐regulating microspheres for ameliorating intervertebral disc degeneration in puncture‐induced degeneration model. (a) The preparation of ClockMPs. (b) Representative scanning electron microscope images of ClockMPs. (c) Representative in vivo imaging images of intervertebral discs at 0, 3, 6, and 9 days after the intervertebral injection of DiR, DiR@Lipo, and DiR@MPs (color scale: Min = 5.77e9). (d) The fluorescence intensity changes with time. (e) The gene expression of Clock in the Control, H_2_O_2_, MPs, Mel, and ClockMPs groups within 72 h. (f) Representative WB results of Clock, Bmal1, and RORα in the Control, H_2_O_2_, MPs, Mel, and ClockMPs groups. (g) Representative WB results of PI3K, p‐PI3K, AKT, and p‐AKT in the Control, H_2_O_2_, MPs, Mel, and ClockMPs groups. (h) Representative Masson staining images of intervertebral discs the five groups at 1, 4, and 8 weeks. (i) Representative X‐ray images of coccygeal intervertebral disc in rats and the IDH change profiles of Healthy, IVDD, MPs, and ClockMPs groups. (j) Representative MRI images of coccygeal intervertebral disc in rats, and the disc gray values were measured to reflect the water content of discs. (k) The gray values of coccygeal intervertebral disc at −1, 1, 4, and 8 weeks after the operation (ClockMPs, circadian clock‐regulating microspheres; DiR, 1,1‐dioctadecyl3,3,3′,3′‐tetramethylindotricarbocyaineiodide; IVDD, intervertebral disc degeneration; Mel, melatonin; MPs, microspheres; Mel@Lipo, liposome loaded with melatonin; MRI, magnetic resonance imaging; NPC, nucleus pulposus cell; PVA, polyvinyl alcohol; PBA, phenylboronic acid).^159^ Copyright, 2023 Wiley.

Polyvinyl alcohol (PVA) is a white, stable, non‐toxic, water‐soluble polymer synthesized through the polymerization and alcoholysis of vinyl acetate.^157^ The high water content of PVA hydrogels allows them to replicate the physiological environment of the IVD, while their superior flexibility and elasticity help maintain the height and mechanical properties of the IVD. For example, a glycerol‐crosslinked PVA gel exhibits storage and compressive moduli comparable to those of the natural NP, along with a porous structure and rapid swelling ability. This gel can sustain the vitality of NPCs under pathological conditions and regulate the ECM metabolic balance.^158^ In a notable study, Chen et al. combined biomaterials with the circadian rhythm of IVD using microfluidic technology to crosslink PVA with modified polybutyl acrylate (PBA), creating a biomaterial that regulates circadian rhythm. This hydrogel microsphere can modulate the expression of core circadian clock genes by activating the PI3K‐AKT pathway, thereby reshaping the intrinsic circadian clock and enhancing ECM synthesis (Figure 7).^159^

Oxidative stress is a major mechanism in IVDD, accelerating IVDD through pathways such as cell damage, matrix degradation, and inflammatory responses.^160^ Feng et al. examined the effects of ROS on IVD by embedding NO‐loaded BioMOFs into ROS‐responsive MPVA/PCL. This composite material can adjust the microenvironment of AF defects by scavenging ROS, inhibiting inflammation, and promoting ECM secretion. Additionally, BioMOFs can continuously release NO to penetrate the dense AF, facilitating cell migration and maintaining ECM homeostasis.^161^ Similarly, a ROS‐responsive hydrogel encapsulating SLC7A11 modRNA has been developed for localized delivery and selective release of modRNA. This modRNA improves IVDD by inhibiting ferroptosis in NPCs.^162^ Furthermore, Bai et al. combined PVA with tsPBA to create a ROS‐scavenging scaffold containing rapamycin. This hydrogel can control the release of rapamycin, effectively scavenging ROS and further reducing inflammatory responses.^163^

#### 6.2.6 Polyacrylamide

Polyacrylamide (PAM) is a high‐molecular‐weight compound, and its derived PAM hydrogel can be used as a filler material for IVD, replacing damaged disc tissue.^164^ This hydrogel exhibits great elasticity and water absorption properties, which aid in restoring the normal elasticity and function of the IVD. Huang et al. employed an inverse emulsion polymerization method to create poly(acrylamide‐co‐acrylic acid) microgels and coated their surfaces with polydopamine to enhance cell adhesion. This hydrogel demonstrates good cell adhesion and biocompatibility and can promote the differentiation of ADSCs into NP‐like cells through static stretching. In vivo, the microgel increases ECM and water content, thus restoring the height and mechanical properties of the IVD.^165^ Similarly, a tri‐crosslinked oxidized HA‐dopamine‐(PAM) composite hydrogel with good adhesion and mechanical properties has been used to deliver TGF‐β1 to promote AF regeneration. This hydrogel recruits AF cells and enhances cell activity through sustained release of TGF‐β1. In vivo, the hydrogel shows good integration with native tissue and supports the repair of the AF.^166^ Additionally, Li et al. developed a bioadhesive made from PAM/alginate/chitosan, where the adhesive is used to fill the NP cavity, and the sealant is employed to close defects in the AF. The results indicate that the adhesive tightly bonds with the IVD tissue and matches the mechanical properties of the NP, effectively withstanding significant IVD loads.^167^

### 6.3 Extracellular matrix/decellularized tissue matrix

ECM hydrogels are designed to mimic the natural ECM. They replicate the natural matrix of the IVD, offering similar mechanical support and stability.^168^ These hydrogels create an environment akin to natural tissues, which promotes cell adhesion and proliferation. Additionally, they possess great water retention properties, helping to maintain the water content of the IVD and prevent dehydration. They also modulate local inflammatory responses, reducing inflammatory damage during degeneration, and serve as scaffolds for cell migration and tissue regeneration.^29^, ^48^ In contrast, DTM is created by removing cellular components from tissues while preserving the three‐dimensional structure and ECM components of the original tissue, such as collagen and glycosaminoglycans.^169^ By eliminating cellular components, DTM minimizes the risk of immune responses, thereby enhancing biocompatibility. DTM hydrogels hold significant promise in tissue engineering and regenerative medicine by providing an environment that closely resembles natural tissue, thereby effectively supporting tissue repair and regeneration.^170^, ^171^

Thanks to the outstanding biocompatibility of ECM and DTM hydrogels, numerous researchers have integrated diverse stem cells into these hydrogels, creating a three‐dimensional support that mimics the in vivo conditions to foster stem cell growth, differentiation, and function.^172^ Ishiguro et al. devised a scaffold‐free tissue engineering construct from adipose‐derived mesenchymal stem cells. Six weeks post‐implantation into the IVD, the construct retained the disc height, endplate, and AF integrity. After 6 months, the construct demonstrated a significant reduction in age‐related biomechanical degeneration of the IVD, showcasing the regenerative prowess of the engineered construct.^173^ Furthermore, Luo et al. incorporated Sphk2‐overexpressing endplate chondrocyte stem cells into rib cartilage ECM‐modified hydrogels. These hydrogels secreted Sphk2‐engineered exosomes capable of penetrating the AF, stimulating NPCs autophagy, and thereby achieving a non‐invasive IVDD therapeutic approach.^174^ Yu et al. employed DTM hydrogels to deliver ADSCs for IVDD mitigation. The hydrogel possessed an elastic modulus akin to human NP and facilitated the expression of NP‐associated genes. Post‐injection, this delivery mechanism enhanced disc height, MRI indices, and histological grading scores.^175^ To ascertain the influence of tissue specificity on stem cell lineage, Peng et al. developed DTM hydrogels from NP and AF, and loaded these with hBMSCs. The findings revealed that NP DTM hydrogels facilitated hBMSC differentiation into NP‐like cells, whereas AF DTM hydrogels induced their differentiation into AF‐like cells, underscoring the intimate link between stem cell differentiation and the native microenvironment.^176^

In addition to cell delivery, ECM and DTM hydrogels are also frequently used for delivering cellular secretions, specifically EVs. Zhang et al. developed DTM hydrogels to encapsulate EVs derived from M2 macrophages. These hydrogels can gradually release EVs, which inhibit pyroptosis in NPCs through miR‐221‐3p transfection. This process promotes tissue regeneration and delays IVDD in vivo.^177^ Liao et al. created a thermo‐responsive hydrogel composed of Pluronic F127 and DTM for extracellular vesicles delivery. This hydrogel enhances the proliferation and migration of NPCs and stimulates the synthesis and metabolism of ECM.^178^ Xing et al. loaded thermo‐sensitive ECM hydrogels with EVs, a specific type of EVs with a diameter of approximately 30–150 nm. The hydrogel provides sustained release of EVs, regulates ECM synthesis and degradation, and alleviates inflammation, thus improving the IVD microenvironment.^179^

Hydrogels, as macroscopic delivery systems, are often combined with nanoparticle delivery systems to create multi‐scale delivery systems. Peng et al. used microfluidic technology to incorporate lactate oxidase‐MnO_2_ nanocatalysts into NP DTM hydrogel microspheres. These microspheres can gradually release lactate oxidase to consume lactate and use nanocatalysts to promote autophagy in NPCs, thereby enhancing cell survival and ECM regeneration.^180^ Additionally, Peng et al. designed a novel multi‐scale delivery system by incorporating amphiphilic cationic nanomicelles into a decellularized AF matrix, resulting in a new composite hydrogel. This hydrogel effectively modulates the inflammatory environment, inhibits nerve growth and sensitization, and shows promise for the clinical treatment of LBP.^181^

## 7 ADVANTAGES AND DISADVANTAGES OF NATURAL AND SYNTHETIC HYDROGELS

Classified by composition source, hydrogels can be categorized into natural hydrogels (such as HA, collagen/gelatin, alginate, chitosan, polypeptides, fibrin, and chondroitin sulfate), synthetic hydrogels (such as gelatin methacrylate, polyethylene glycol, polycaprolactone, polylactic acid/polyglycolic acid, polyvinyl alcohol, and polyacrylamide), and the ECM.

Natural‐component hydrogels, sourced from biomaterials, demonstrate exceptional compatibility with human tissues, effectively mitigating immune responses and inflammation while fostering tissue healing. For instance, Wang et al.'s nanostructured gelatin colloidal hydrogel not only stimulates MSC proliferation but also sustains cell viability above 95%.^77^ Additionally, these hydrogels typically exhibit great biodegradability, breaking down gradually within the body into metabolites that are predominantly non‐toxic and do not accumulate, thereby minimizing long‐term usage risks. Chen et al.'s zinc‐oxidized sodium alginate‐gelatin hydrogel, for example, degrades into amino acids and sugars within about 2 weeks.^84^ Moreover, natural hydrogels can emulate the ECM, promoting cell adhesion, proliferation, and differentiation, thereby facilitating tissue regeneration. The BMSC‐loaded photo‐crosslinked HA/collagen hydrogel has proven effective in enhancing BMSC proliferation and differentiation.^62^ By fine‐tuning the concentration and crosslinking degree of natural hydrogels, their mechanical properties can be tailored to diverse application requirements. For instance, Koo et al. synthesized a collagen scaffold with shape memory capabilities by precisely evaluating the hydrogel's concentration and crosslinking degree.^70^ Furthermore, natural component hydrogels can be readily chemically modified to incorporate functional groups, expanding their capabilities, such as integrating drug carriers, growth factors, and other functional entities. The extraction and processing methods for these hydrogels are well‐established and characterized by straightforward procedures and relatively low costs. Notably, many natural component hydrogels exhibit multifunctionalities, including HA's moisture retention, collagen's structural support, and sodium alginate's ionic crosslinking properties.

Certainly, while natural hydrogels possess distinct advantages, they are also accompanied by certain shortcomings. Natural‐component hydrogels frequently demonstrate relatively low mechanical strength, rendering them incapable of enduring high stress and extended usage, thereby limiting their applicability in specific high‐load tissue repairs. The chitosan and HA crosslinked nano‐composite hydrogel, though possessing various biological functions, has a storage modulus of only 100 Pa and a loss modulus of less than 10 Pa, which is inadequate for adapting to the high‐pressure environment within the IVD.^98^ Such characteristics render it ill‐suited for the high‐pressure environment within the IVD. Certain natural‐component hydrogels are deficient in sufficient antibacterial efficacy, requiring the incorporation of antibacterial agents or the implementation of surface antibacterial coatings when used in the body, thus escalating complexity and expense.^76^, ^80^ Owing to the biological heterogeneity inherent in the sourcing of natural‐component hydrogels, material properties may diverge between different batches, affecting the consistency and reliability of the product. Moreover, the intricate chemical constitution of natural‐component hydrogels presents formidable challenges during processing, encompassing issues of solubility and stability, thereby circumscribing their potential to be fashioned into intricate structures.

Synthetic hydrogels generally possess higher mechanical strength and elastic modulus compared to natural component hydrogels, enabling them to withstand greater stress and strain, making them suitable for tissue repair requiring higher mechanical support, such as the IVD and other bone joints. Cheng et al. designed a hydrogel with high mechanical strength, featuring a storage modulus of up to 10^4^ Pa and a loss modulus of approximately 10^3^ Pa.^182^ Additionally, the chemical composition, crosslinking degree, degradation rate, and porosity of synthetic hydrogels can be precisely regulated through the synthesis process, allowing them to be customized according to specific application requirements. Jia et al. developed a glycerol crosslinked PVA gel that exhibits injectability, NP‐like viscoelastic characteristics, great energy dissipation properties, and swelling ability.^158^ Regarding degradation, the degradation rate of synthetic hydrogels can be adjusted by altering the polymer's chemical structure and crosslinking degree, enabling precise control over the material's metabolic rate within the body. The degradation time can range from a few days to several weeks or even months.^130^, ^165^, ^183^ Furthermore, synthetic hydrogels can be easily prepared into complex shapes and structures through various processing techniques, such as 3D printing and microforming, enabling personalized customization to meet different anatomical needs. Since the synthesis process is conducted under controlled conditions, synthetic component hydrogels exhibit smaller batch‐to‐batch variability, resulting in higher product stability and consistency, facilitating large‐scale production and application. By chemically modifying and introducing various functional groups, such as cell adhesion sites, drugs, and growth factors, synthetic hydrogels can be endowed with multifunctionality to meet diverse therapeutic demands.

Compared to natural component hydrogels, synthetic hydrogels generally lack natural bioactivity and may not effectively promote cell adhesion, proliferation, and differentiation, necessitating additional modifications to introduce bioactive molecules. Some synthetic hydrogels may exhibit inferior biocompatibility compared to natural component hydrogels, potentially triggering immune or inflammatory responses, especially during long‐term implantation. Although certain synthetic hydrogels possess strong resistance to degradation, this may lead to prolonged material presence within the body in some cases, increasing the risk of long‐term implantation issues such as fibrosis or chronic inflammation. Additionally, synthetic hydrogels typically cannot fully mimic complex biological microenvironments, such as the porous structure and multifunctionality of the ECM, which may limit their application in certain complex tissue repairs.

## 8 CLASSIFICATION OF HYDROGEL‐DELIVERED SUBSTANCES

**FIGURE 8:**
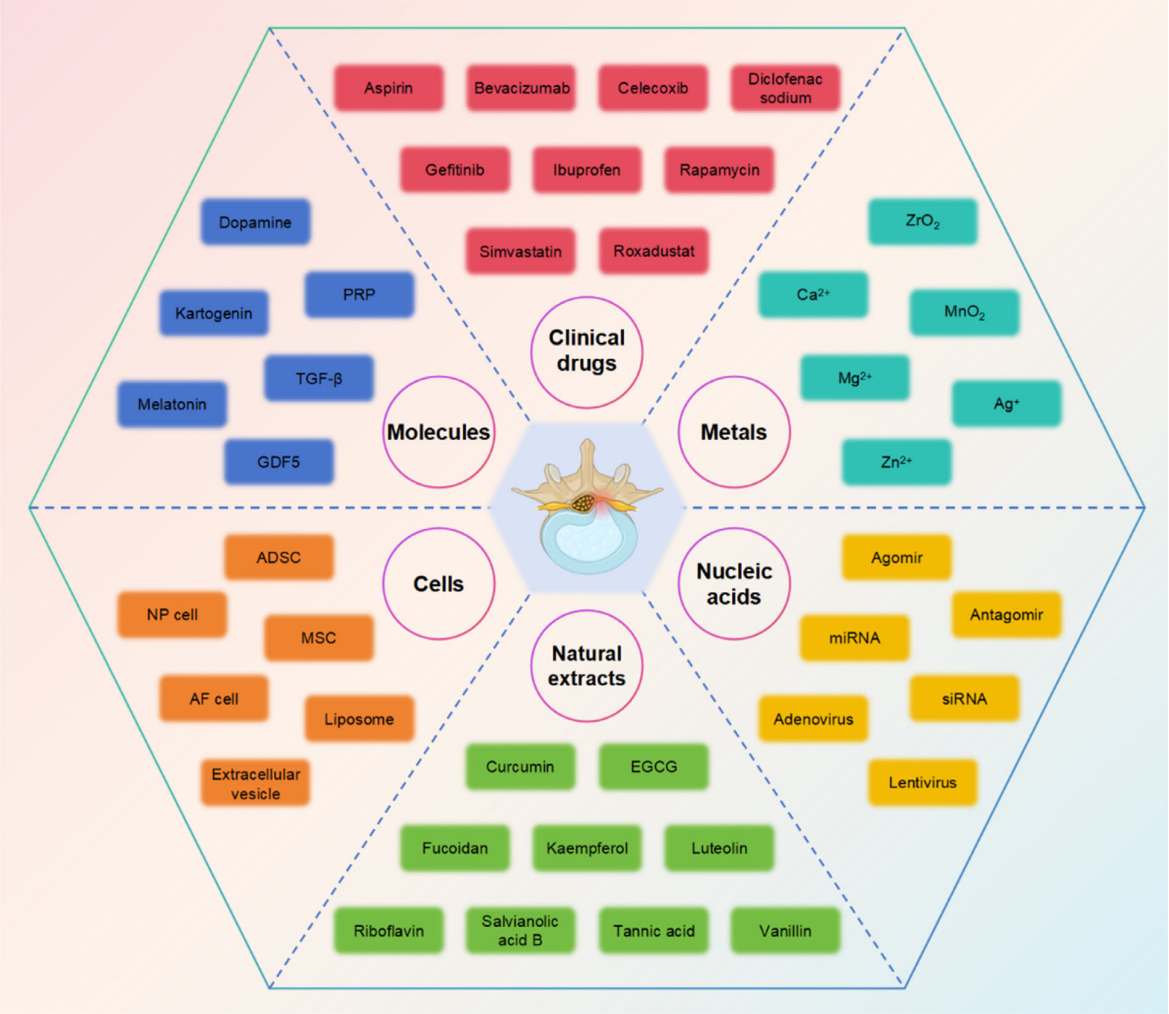
Classification of substances delivered by hydrogels: molecules, clinical drugs, metals, nucleic acids, natural extracts, and cells (ADSC, adipose derived stem cell; AF, annulus fibrosus; EGCG, epigallocatechin‐3‐gallate; GDF5, growth differentiation factor 5; MSC, mesenchymal stem cell; NP, nucleus pulposus; PRP, platelet‐rich plasma; TGF‐β, transforming growth factor‐β). Created with www.BioRender.com.

Hydrogels, as versatile delivery systems, are increasingly attracting attention for their applications in the treatment of disc degeneration. By encapsulating and delivering various types of substances, hydrogels can play different roles in the therapy of disc degeneration. The delivered substances can be broadly categorized into six types: clinical drugs, metals, nucleic acids, natural extracts, cells, and molecules (Figure 8). The clinical drugs delivered by hydrogels include aspirin, celecoxib, gefitinib, and others. Hydrogels can control the drug release rate, reduce the drug dosage, and minimize side effects. Metal substances such as Zn^2+^, Ag^+^, and Ca^2+^ can be delivered via hydrogels to promote wound healing and antibacterial properties. Precise control over metal ion delivery is necessary to avoid toxicity and ensure therapeutic efficacy. The stability and controllable degradation of hydrogels provide an ideal platform for metal ion delivery. Nucleic acid substances, including miRNA, siRNA, and lentivirus, can be protected from enzymatic degradation, enhance their stability in the body, and achieve targeted delivery through hydrogel delivery systems. This is crucial for applications such as gene silencing, gene editing, and protein expression regulation. Natural extracts such as curcumin, kaempferol, and luteolin can have their bioactivity protected, their half‐lives extended in the body, and their bioavailability improved by hydrogels. Cell therapy, particularly stem cell therapy, is at the forefront of regenerative medicine. Hydrogels can serve as three‐dimensional culture systems for cells, providing necessary nutrients and growth factors to promote cell survival, proliferation, and differentiation. Molecules such as PRP, TGF‐β, and dopamine can be delivered through hydrogel systems to achieve targeted and controlled release.

## 9 CONCLUSIONS AND FUTURE PERSPECTIVES

**FIGURE 9:**
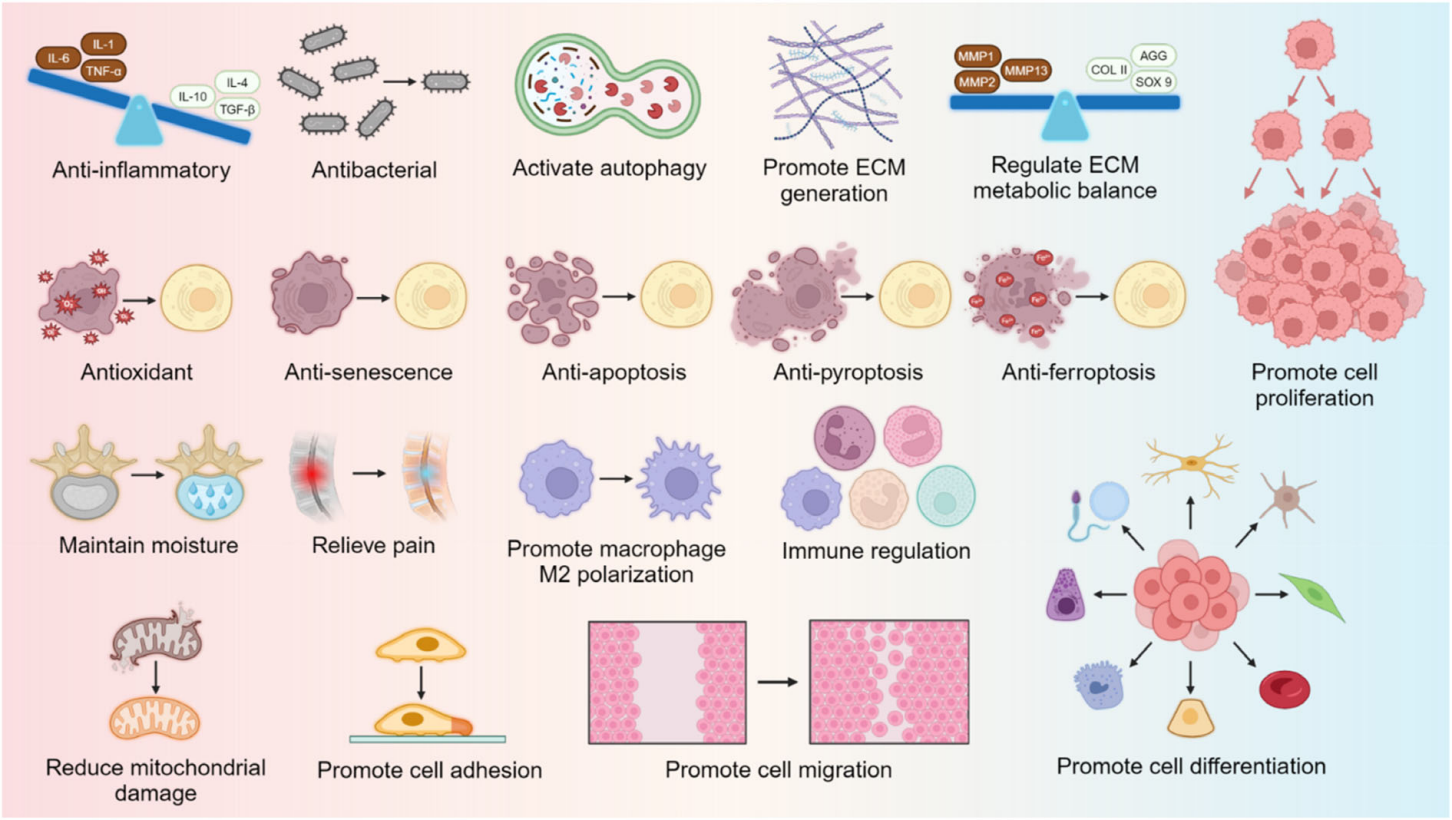
Schematic illustration of the biological features of hydrogels. The biological properties of hydrogels: anti‐inflammatory, antibacterial, activate autophagy, promote ECM generation, regulate ECM metabolic balance, antioxidant, anti‐senescence, anti‐apoptosis, anti‐pyroptosis, anti‐ferroptosis, promote cell proliferation, maintain moisture, relieve pain, promote macrophage M2 polarization, immune regulation, reduce mitochondrial damage, promote cell adhesion, promote cell migration, and promote cell differentiation (ECM, extracellular matrix). Created with www.BioRender.com.

**FIGURE 10:**
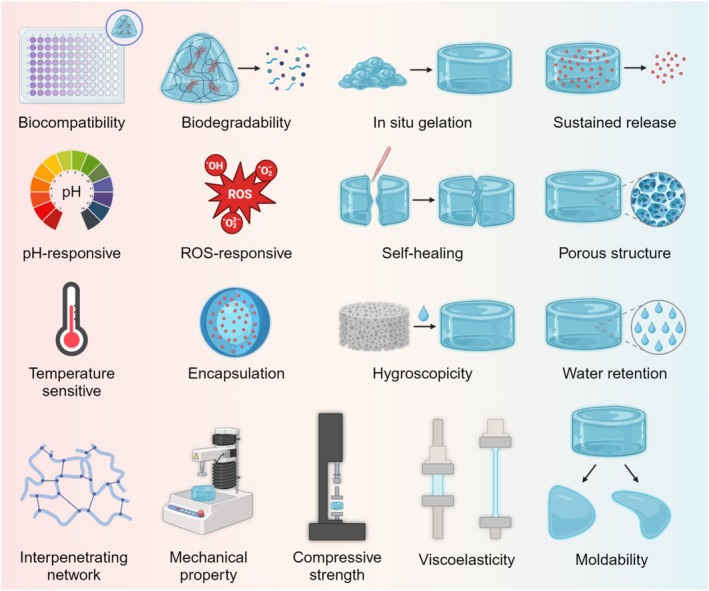
Schematic illustration of the material features of hydrogels. The material properties of hydrogels: biocompatibility, biodegradability, in situ gelation, sustained release, pH‐responsive, ROS‐responsive, self‐healing, porous structure, temperature sensitive, encapsulation, hygroscopicity, water retention, interpenetrating network, mechanical property, compressive strength, viscoelasticity, and moldability (ROS, reactive oxygen species). Created with www.BioRender.com.

This review gives a brief overview of the physiology of IVD and the pathophysiology of IVDD, including its diagnostic methods and current treatment choices. Notably, it explores recent advances in using hydrogels for treating IVDD, focusing on the biological and material characteristics of different hydrogel types (Figures 9 and 10). Moreover, it discusses the benefits and drawbacks of both natural and synthetic hydrogels and summarizes the classification of hydrogel delivery substances. In situ crosslinking injectable hydrogels demonstrate outstanding mechanical properties, biocompatibility, biodegradability, and drug delivery capabilities, positioning them as a potent tool for IVDD treatment. Notably, the three‐dimensional crosslinking network structure of the hydrogel, along with its controllable physical and chemical properties, allows for the mimicry of the IVD's structure and mechanical properties through alterations in material types, molecular weights, and degree of crosslinking. This makes these hydrogels particularly well‐suited for the regeneration and repair of the IVD.

Although hydrogels have shown great potential in the treatment of IVDD, they still face numerous challenges in practical applications. For example, the delivery of cells encapsulated in hydrogels for IVDD treatment faces challenges related to anoikis, which is the apoptosis of cells in non‐adherent environments.^184^ This issue is particularly problematic in degenerated discs, where the combined effects of inflammation, hypoxia, and acidic conditions severely impact cell survival and function. Additionally, hypoxia within the IVD and accelerated cell death make it difficult for drugs and other bioactive factors to exert their effects. Although the mechanical properties of some hydrogels have been demonstrated to be great in vitro experiments, most mechanical performance tests are still limited to in vitro environments, and many in vivo models use small animals such as mice, rats, and rabbits. These experimental results are still a long way from practical application in humans. More importantly, there may be some contradictions between the material properties and biological performance of hydrogels. For example, the contradiction between the mechanical properties and biocompatibility of hydrogels. The high water content characteristic limits the number of macromolecules available within the hydrogel to resist external forces, resulting in hydrogel materials being inherently soft and weak. Another example is the contradiction between the strength and toughness of hydrogels. In materials science, strength and toughness are often difficult to balance, and this contradiction is particularly prominent in hydrogel materials. Hydrogels need to maintain high strength while also possessing good toughness, which presents a significant challenge to the preparation technology of hydrogels.^185^

Future research on hydrogels might focus on the following directions: Firstly, optimize the mechanical properties of hydrogels through multiscale simulations and composite material design to enhance their strength and elasticity. Secondly, augment the biological activity of hydrogels by incorporating bioactive molecules that promote cell growth and tissue regeneration. Additionally, conduct large animal models and long‐term in vivo experiments to verify the safety and long‐term effects of hydrogels. Through in‐depth exploration of these research directions, it is expected to further enhance the application of hydrogels in IVDD treatment, providing patients with safer and more effective treatment options.

In summary, hydrogels show great potential in treating IVDD due to their biocompatibility, injectability, and tissue repair capabilities, making them ideal for disc repair materials. However, the mechanical properties and long‐term biological activity of existing hydrogel materials still need improvement. Therefore, continued research and development of new hydrogel materials, simultaneously enhancing their biological and material properties, hold significant promise for IVDD treatment.

## AUTHOR CONTRIBUTIONS

Tao Chen: Data curation; Investigation; Software; writing – original draft. Dading Lu: writing – review & editing; Investigation. Siqiao Wang: writing – review & editing; Software. Huiyi Yang: writing – review & editing; Investigation. Wenyong Fan: Validation. Zhihui Xiao: Conceptualization. Zhaojie Wang: Methodology. Pooyan Makvandi: writing – review & editing; Investigation; Supervision. Rongrong Zhu: Supervision; Funding acquisition. Liming Cheng: Validation; Supervision; Resources; Funding acquisition; Conceptualization.

## FUNDING INFORMATION

National Key Research and Development Program of China, Grant/Award Number: 2016YFA0100800; International (Regional) Cooperation and Communication Program of the National Natural Science Foundation of China, Grant/Award Number: 81820108013; Shanghai Key Discipline Clinical Research Center Construction Program, Grant/Award Number: 2023ZZ02016; National Key Clinical Specialty Discipline Construction Program of China, Grant/Award Number: Z155080000004; Key Program of National Natural Science Foundation of China, Grant/Award Number: 82330062.

## CONFLICT OF INTEREST STATEMENT

The authors declare no conflicts of interest.

## Data Availability

Data sharing is not applicable to this article as no new data were created or analyzed in this study.
